# Risk of glaucoma to subsequent dementia or cognitive impairment: a systematic review and meta-analysis

**DOI:** 10.1007/s40520-024-02811-w

**Published:** 2024-08-20

**Authors:** Xiaoran Wang, Wenjing Chen, Wenxia Zhao, Mingsan Miao

**Affiliations:** 1 Department of Clinical, Henan University of Chinese Medicine, No.156 Jinshui East Road, Zhengzhou, Henan 450046 China; 2 Department of Pharmacology, Henan University of Chinese Medicine, No.156 Jinshui East Road, Zhengzhou, Henan 450046 China; 3 The First Affiliated Hospital, Henan University of Chinese Medicine, No. 19 Renmin Road, Jinshui District, Zhengzhou, Henan 450003 China; 4 National International Cooperation Base of Chinese Medicine, Henan University of Chinese Medicine, Zhengzhou, China

**Keywords:** Glaucoma, Dementia, Alzheimer’s disease, Cognitive impairment, Meta-analysis

## Abstract

**Background:**

Substantial evidence supports that glaucoma and dementia share pathological mechanisms and pathogenic risk factors. However, the association between glaucoma, cognitive decline and dementia has yet to be elucidated.

**Objective:**

This study was aimed to assess whether glaucoma increase the risk of dementia or cognitive impairment.

**Methods:**

PubMed, Cochrane Library, Web of Science, and EMBASE databases for cohort or case-control studies were searched from inception to March 10, 2024. The Newcastle-Ottawa Quality Assessment Scale (NOS) was used to the risk of bias. Heterogeneity was rigorously evaluated using the *I*^2^ test, while publication bias was assessed by visual inspection of the funnel plot and by Egger’ s regression asymmetry test. Subgroup analyses were applied to determine the sources of heterogeneity.

**Results:**

Twenty-seven studies covering 9,061,675 individuals were included. Pooled analyses indicated that glaucoma increased the risk of all-cause dementia, Alzheimer’s disease, vascular dementia, and cognitive impairment. Subgroup analysis showed that the prevalence of dementia was 2.90 (95% CI: 1.45–5.77) in age ≥ 65 years and 2.07 (95% CI: 1.18–3.62) in age<65 years; the incidence rates in female glaucoma patients was 1.46 (95% CI: 1.06-2.00), respectively, which was no statistical significance in male patients. Among glaucoma types, POAG was more likely to develop dementia and cognitive impairment. There were also differences in regional distribution, with the highest prevalence in the Asia region, while glaucoma was not associated with dementia in Europe and North America regions.

**Conclusion:**

Glaucoma increased the risk of subsequent cognitive impairment and dementia. The type of glaucoma, gender, age, and region composition of the study population may significantly affect the relationship between glaucoma and dementia.

**Supplementary Information:**

The online version contains supplementary material available at 10.1007/s40520-024-02811-w.

## Introduction

Dementia, a growing global public health problem, affects approximately 50 million people. As life expectancy rises, the number of dementia cases worldwide is expected to skyrocket to more than 131 million by 2050 [1]. As a neurodegenerative disease, the widespread prevalence of dementia places a significant strain on global healthcare systems. Due to the lack of effective treatments and preventive interventions, identifying potential risk factors for dementia is critical for dementia prevention. However, no disease-modifying treatments are currently available for adults with dementia; thus, an emphasis on risk factor reduction, particularly modifiable risk factors, is warranted. According to recent research, visual impairment is one of the first symptoms of dementia [2]. Visual deprivation caused by retinal ganglion cell (RGC) injury may result in decreased activation of central sensory pathways in the brain, resulting in decreased cognitive load and an increased risk of structural brain damage, accelerating the progression of dementia [3, 4].

Glaucoma is a group of diseases characterized by optic papillary atrophy and depression, as well as retinal ganglion cell (RGC) death and visual field defects, which is the most common cause of irreversible blindness worldwide. Clinically, it is classified as open-angle glaucoma or angle-closure glaucoma based on the status of the anterior chamber angle at the time of elevated intraocular pressure [5, 6]. Despite extensive multicenter and laboratory studies showing that pathological intraocular pressure (IOP) elevation is a significant risk factor for the development and progression of the disease, lowering IOP does not always stop the disease [7]. In glaucoma patients, progressive loss of visual function is associated with RGC degeneration, characterized by apoptosis of retinal somatic cells, axonal degeneration of the optic nerve, and synaptic loss of dendrites and synaptic loss of axon terminals. In addition, glaucoma-related neuronal damage extends to the lateral geniculate nucleus and visual cortex and is accompanied by astrocyte and retinal microglia changes [8–10]. Axonal transport defects have also been linked to several neurodegenerative diseases, including Alzheimer’s disease (AD) and other dementias. Although inconclusive, numerous research have been conducted to support the common pathophysiological features of dementia and glaucoma regarding age-related biological features and cell death mechanisms in the central nervous system [11].

Several cross-sectional studies have found that glaucoma is associated with deficits in a variety of cognitive functions, including attention, language, learning, and memory skills [12–15]. In a study of 1,168 elderly patients, Mandas et al. found a significant association between glaucoma and the prevalence of mixed dementia [16]. Furthermore, neuropathological studies have revealed hyperphosphorylated tau proteins, increased amyloid fragmentation, microglia activation, neurodegeneration, and apoptosis in the retinas of glaucoma patients. However, existing evidence does not explain the causal relationship between glaucoma and dementia or cognitive impairment, and findings are inconsistent [17–19]. For example, in a cross-sectional study in Denmark, Bach-Holm et al. followed 69 elderly patients with normal IOP glaucoma for 12 years and found no correlation between glaucoma and dementia [20]. Ong et al. followed 1179 older adults with age-related eye disease in Singapore and suggested no significant correlation [21]. The relationship between glaucoma and dementia or cognitive impairment remains controversial.

Growing evidence suggests that glaucoma and dementia share pathological mechanisms and pathogenic risk factors. Nevertheless, previous studies reported inconsistent results regarding the association between glaucoma and dementia or cognitive impairment [22]. Clarifying the effects of glaucoma on subsequent secondary cognitive impairment or dementia is critical for preventing and delaying the progression of these diseases. Therefore, we performed a systematic review and meta-analysis to assess the association of glaucoma with dementia or cognitive impairment.

## Methods

This current systematic review conformed to the Preferred Reporting Items for Systematic Reviews and Meta-Analyses (PRISMA) guidelines [23] and was registered in the International Prospective Register of Systematic Reviews: https://www.crd.york.ac.uk/ PROSPERO (Registration number: CRD 42,023,408,202).

### Search strategy

Both Medical Subject Headings (MeSH) terms and keywords were utilized to retrieve as many as possible in PubMed, Web of Science, EMBASE, and Cochrane Library for case-control or cohort studies exploring the relationship between glaucoma and dementia or cognitive impairment published from their inception to March 10, 2024. The main terms included “glaucoma,” “dementia,” “Alzheimer’s disease,” “vascular dementia,” “senile dementia,” “cognitive decline,” “cognitive impairment,” “cognitive dysfunction,” and “cognitive disorder.” A further gray literature search was conducted using Google Scholar to identify relevant articles not found through the database search. References and citations of relevant publications identified for inclusion and reviews on this topic were scrutinized. English and Chinese language publications were included. The detailed search strategy is presented in Supplementary Table 1.

### Eligibility criteria

The included studies were required to meet the following criteria: (1) case-control or cohort study design; (2) exposure factors were glaucoma with incident dementia or cognitive impairment, and the control group included participants without glaucoma; (3) report risk estimates of dementia or cognitive decline as the outcome (i.e., at least all-cause dementia or Alzheimer’s disease as its most common subtype), expressed as an adjusted odds ratio (OR), risk ratio (RR), or hazard ratio (HR); (4) population-based study design; (5) English publication.

### Exclusion criteria

The exclusion criteria were as follows: (1) conference abstract, reviews, case reports, basic experiments and other non-clinical research; (2) duplicate publications; (3) studies with incomplete data or no relevant outcome.

### Study selection and data extraction

All retrieved records were imported into an EndNote (Clarivate Analytics) library, and two researchers (WXR and CWJ) independently screened the literature, extracted data, and cross-checked. Discussions were cross-verified by a third researcher in the event of disagreement. When screening literature, first read the title and abstract, and after excluding irrelevant literature, further, read the full text to determine what is ultimately included. (1) Basic information: first author, publication year, country, study type; (2) Characteristics of included studies: sample size, gender, age, follow-up years, diagnostic criteria for glaucoma, dementia, or cognitive impairment, and adjusted covariates; (3) Key elements of risk of bias assessment; and (4) Effect sizes and their 95% confidence intervals (CI) after controlling for confounding factors.

### Data synthesis

Following the PRIMA 2020 guidelines, the selection process was documented using a “flow diagram” showing the number of references excluded at each step. Reasons for study exclusion after full-text assessment are reported in detail. In addition, the extracted data were tabulated and summarized in text. Moreover, the results of the statistical analysis are presented in both tables and figures (detailed below).

### Assessment of risk of bias

To determine the validity of the included studies, two independent investigators assessed the risk of bias (RoB) using the Newcastle-Ottawascale Scale (NOS) [24] and cross-checked the results. In the case of any disagreement, a third party was consulted to assist in the decision. The evaluation was conducted in three parts with eight items, with scores ranging from 0 to 4 indicating low quality, 5 to 6 indicating medium quality, and 7 to 9 indicating high quality. Each study was assigned a risk of bias rating - high, moderate, or low - based on responses to each question.

### Statistical analysis

All statistical analyses were conducted by using RevMan 5.3 and State 17.0 software. The primary outcome indicators were the pooled odds ratios (OR) and 95% confidence interval (95% CI) with adjusted confounders. We formally assessed between-study heterogeneity (chi-square test, α = 0.1) to determine the share of variation across studies due to heterogeneity rather than chance (Higgins’ I^2^ statistic) and interpreted heterogeneity as potentially insignificant (40%), moderate (30-60%), significant (50-90%), or considerable (75-100%), in line with Cochrane recommendations [25]. If *P* > 0.05 or I^2^ < 50%, there was no statistical heterogeneity between studies, and a fixed-effects model was selected. When clinical or statistical heterogeneity occurred, a random-effects meta-analysis was used. An α-level of 0.05 was used to determine statistical significance. We visually examined funnel plot asymmetry and performed Egger’s regression test to detect statistical publication bias (Lin et al., 2018). To confirm the robustness of the overall results, we performed a sensitivity analysis by rerunning the meta-analysis while omitting one study at a time, or by trim-and-fill method. Given that the study region, type of glaucoma, type of dementia, gender, age, sample size, and follow-up time could affect the findings of the study, we performed subgroup analyses to explore sources of heterogeneity.

## Results

### Literature search

**Fig. 1:**
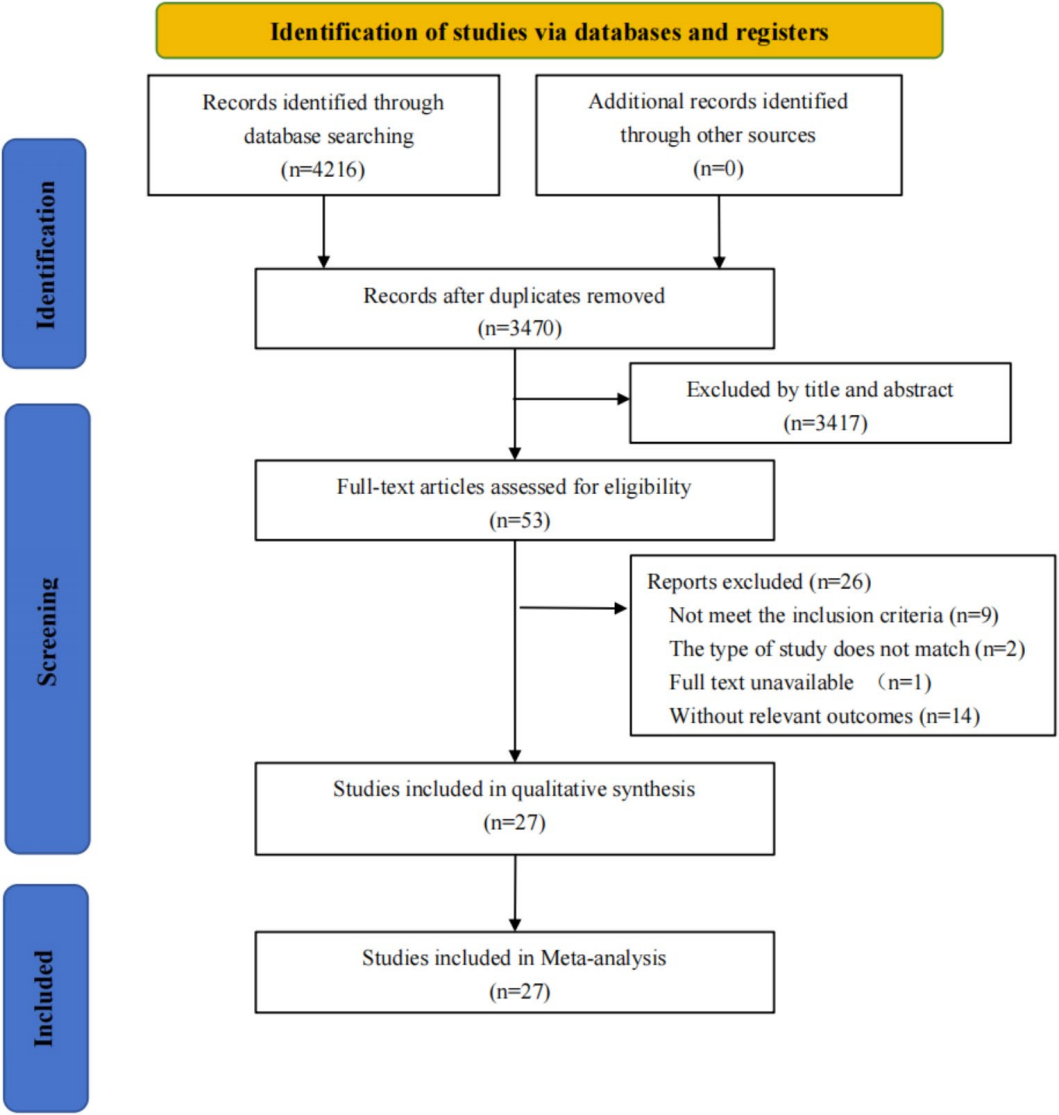
PRISMA flow diagram of the study selection process

In summary, the search retrieved 4,216 records from electronic databases. No additional articles were included based on reference screening and expert consultation. After removing duplicates, 3,467 publications were initially screened by reading the titles and abstracts of the literature. Using the above inclusion/exclusion criteria, the full texts of 54 articles were evaluated, and 27 studies were eventually included. There were twenty cohort studies [26–45] and seven case-control studies [46–52] involving 9,061,675 participants (Fig. 1). The detailed exclusion list is provided in Supplementary Table 2.

### Main characteristics of included studies

Across the included studies, there were 9,061,675 participants aged 18 years or older, with the proportion of male participants ranging from 35.01 to 54.66%. The study populations were from China (*n* = 8) [33, 34, 36, 40, 42, 48, 49, 51] the United States (*n* = 5) [31, 32, 38, 39, 46], Sweden (*n* = 5) [26, 30, 44, 45, 50], Korea (*n* = 3) [27, 28, 35], the United Kingdom (*n* = 2) [28, 37], Australia (*n* = 1) [52], France (*n* = 2) [43, 47], and Denmark (*n* = 1) [41]. These articles were published from 2007 to 2024, with sample sizes ranging from 509 to 2,623,130, and the mean follow-up time varied from 3 to 14 years.

Most of these studies used the International Classification of Diseases-9 (ICD-9) or International Classification of Diseases-10 (ICD-10) diagnosis codes as glaucoma and dementia diagnostic criteria. However, seven studies [30, 31, 43, 44, 45,, 51, 52] used ophthalmologic examinations to assess glaucoma, one [36] used self-administered questionnaires only to determine glaucoma, and four [32, 36, 51, 52] used MMSE/McOA scores to diagnose dementia and cognitive impairment. All studies examined the association between glaucoma and dementia or cognitive impairment as a dichotomous variable among the outcome indicators. In 24 studies [26–45, 47, 50], the outcome measures were dementia and cognitive impairment in three [46, 51, 52]. The main characteristics of the included studies are summarized in Table 1.

**Table 1:** Basic characteristics of the included studies

Authors	Country	Study design	Sample size (exposed/ control)	Age (years)	Follow-up years	Diagnosis of glaucoma	Diagnosis of dementia /cognitive impairment	Glaucoma type	Outcome	Variables adjusted
Crump et al. 2024	Sweden	Retrospective cohort study	32,339/ 226,896	-	22	ICD−9/ICD−10	ICD-9/ ICD-10	POAG/ PACG	All-cause dementia /AD/VD	age, sex, birth country, education, income, hypertension, diabetes, hyperlipidemia, ischemic heart disease, and Charlson Comorbidity Index at index date
Kim et al. 2023	Korea	Prospective cohort study	875/ 3,500	≥ 55	8	ICD-10	ICD-10	POAG/ PACG	All-cause dementia /AD	age, gender, sex, residence, and household income
Park et al. 2023	Korea	Retrospective cohort study	788,961	≥ 45	10.9 ± 2.7	ICD-10	ICD-10	Glaucoma	All-cause dementia /AD/VD	age, sex, and income level, diabetes, hypertension, dyslipidemia, stroke, chronic heart disease, depression, smoking status, drinking habits, body mass index, diabetic retinopathy, age-related macular degeneration, and visual acuity
Shang et al. 2023	UK	Retrospective cohort study	6,386/ 87,602	63.4 ± 4.0/ 62.0 ± 4.0	10.7–11.7	ICD-10	ICD-10	Glaucoma	All-cause dementia	age, gender, education, income, cooked vegetables intake, raw vegetables intake, fresh fruits intake, dried fruits intake, smoking, alcohol consumption, physical activity, BMI, cholesterol and glucose
Ekström et al. 2021	Sweden	Retrospective cohort study	264/ 1,269	65–74	13	Perimetry	ICD-10	POAG	AD	age, a time-dependent variable, and competing events
Belamkar et al.2021	UAS	Retrospective cohort study	509	67.5	10	Eye examination with IOP	DSM-5	POAG	AD	
Hwang et al. 2021	USA	Retrospective cohort study	601/ 571	75.2 ± 4.8/ 74.4 ± 4.9	6–8	ICD-9	MRI/ MMSE	Glaucoma	All-cause dementia /AD/VD	age, sex, race, education, cardiovascular and dementia risk factors, smoking status, alcohol intake, physical activity level, total cholesterol level, diabetes mellitus status, hypertension statue. and cardiovascular diseases
Su et al. 2016	China	Retrospective cohort study	6,509/ 26,036	59	10	ICD-9-CM	ICD-9-CM	POAG、 PACG	All-cause dementia	age, gender, hypertension, diabetes, coronary heart disease, hyperglycaemia, and head injury
Chen et al. 2018	China	Retrospective cohort study	15,317/ 61,268	62.1 ± 12.5	4.92 ± 3.29	ICD-9-CM	ICD-9	POAG (NTG)	AD	age, gender, hypertension, diabetes, hyperglycaemia, coronary artery disease, and stroke
Moon et al. 2018	Korea	Retrospective cohort study	1,469/ 7,345	≥ 18	12	KCD	KCD	POAG	AD	age, sex, residential area, income, Charleston comorbidity index, hypertension, diabetes mellitus, hyperglycaemia and ischemic
Xiao et al. 2020	China	Retrospective cohort study	1,062	71.5 ± 7.4	5.2	self-report questionnaire	DSM-IV/ MMSE	Glaucoma	All-cause dementia /AD	age, sex, education year, cigarette smoking, alcohol consumption, hypertension, diabetes mellitus, BMI, depression, and heart
Keenan et al. 2014	UK	Retrospective cohort study	87,658/ 2,535,472	≥ 55	12	ICD-10	ICD-10	POAG	AD/VD	
Lee et al. 2018	USA	Prospective cohort study	3,877	≥ 65	8	ICD-9	NINCDS- ADRDA	Glaucoma	AD	
Ou et al. 2012	USA	Retrospective cohort study	63,325/ 63,325	78.5	14	ICD−9	ICD-9	POAG	All-cause dementia /AD	
Lin et al. 2014	China	Retrospective cohort study	3,979/ 15,916	71.3 ± 7.08/ 71.3 ± 7.41	8	ICD−9-CM	ICD-9-CM	POAG	AD	age, sex, hypertension, diabetes, heart failure, stroke, insurance eligibility group, monthly income, urbanization level and Charleston commodities index
Kessing et al. 2007	Denmark	Retrospective cohort study	410,544	68.2	4.6	ICD-8 or ICD-10	ICD-8 or ICD-10	PACG	All-cause dementia /AD/VD	age, sex, time from discharge, and a diagnosis of substance use
Kuo et al. 2020	China	Retrospective cohort study	21,024/ 21,024	≥ 20	16	ICD−9/ICD−10	ICD-9/ ICD-10	Glaucoma	All-cause dementia /AD/VD	age, sex, education, marry, hypertension, ischemic heart diseases, hyperglycaemia, congestive heart failure, peripheral vascular disease, cerebrovascular disease, and hemiplegia or paraplegia
Helmer et al. 2013	France	Prospective cohort study	8,12	79.7 ± 4.3	3	Eye examination with IOP	DSM-IV	POAG	All-cause dementia	age, sex, education, hypertension, diabetes, history of cardiovascular ischemic disease, history of stroke, familial history of glaucoma, and apolipoprotein
Ekstrom and Kilander 2016	Sweden	Retrospective cohort study	1,533	65–74	30	Eye examination	Medical chart evaluation	POAG	AD	age, gender, participating in the population survey, smoking habits, diabetes mellitus, systemic hypertension, ischemic heart disease
Ekström and Kilander 2014	Sweden	Retrospective cohort study	1,123	65–74	14.0 ± 6.4	Eye examination with IOP	ICD-9	POAG	AD	
Umunakwe et al. 2020	USA	Case-control study	24,892/ 1,484,790	58.9 ± 14.0/ 44.9 ± 14.1	4.7	ICD-9/ICD-10	ICD-9/ ICD-10	POAG	VD/AD/ cognitive impairment	age, race, and gender
Chamard et al. 2023	France	Case-control study	4,810/ 24,050	≥ 60	7	ICD-10	ICD-10	Glaucoma	All-cause dementia	overweight or obesity, diabetes, antihypertensives, hypolipidemic drugs, chronic kidney disease, stroke, coronary heart disease, heart failure, heart rhythm disorders, heart conduction disorders, lower extremity arterial disease, depression or bipolar disorder, psychotic disorders, Diamox, benzodiazepines cDDD levels and anticholinergics cDDD levels.
Chung et al. 2015	China	Case-control study	264/ 15,276	76.8 ± 9.6	6	ICD-9	ICD-9	POAG	All-cause dementia	urbanization level, monthly income, geographic region, hypertension, diabetes, hyperglycaemia, tobacco use disorder, and alcohol abuse, and the number of outpatient visits within 1 year prior to index date
Lai et al. 2017	China	Case-control study	6,680	78.0 ± 6.7/ 78.7 ± 6.6	11	ICD-9	ICD-9	Glaucoma	AD	age
Wändell et al. 2022	Sweden	Case-control study	7,791/ 1,695,884	>18	6	ICD-10	ICD10	POAG	AD	
Wang et al. 2021	China	Case-control study	116/2,959	69.42 ± 6.77	-	Eye examination	MMSE /MoCA	Glaucoma	Cognitive impairment	
Mullany et al. 2022	Australia	Case-control study	144/146	≥ 65	-	Eye examination with IOP	T- MoCA	POAG	Cognitive impairment	

*Abbreviations* POAG: primary open-angle glaucoma; PACG: primary angle-closure glaucoma; AD: Alzheimer’s disease; VD: vascular dementia; SD: senile dementia; ICD-8/9/10: International Classification of Diseases, version 8/9/10; MMSE: Mini-mental State Examination; MoCA: Montreal Cognitive Assessment; DSM-IV: Diagnostic Statistical Manual Mental Disorders IV; KCD: Korean Classification of Diseases; NINCDS-ADRDA: National Institute of Neurological and Communicative Disorders and Stroke–Alzheimer’s Disease and Related Disorders Association; MRI: magnetic resonance imaging; IOP: Intra-ocular Pressure; BMI: Body Mass Index

### Methodology quality assessment

The NOS scale was used to assess the quality of the included studies, and the results are presented in Tables 2 and 3. Twenty-one studies [26–36, 38–45, 48, 49] with a quality score ≥ 7 were classified as high quality, five [37, 47, 50–52] with a quality score of 6, and one [46] with a quality score of 5 was classified as moderate quality. In terms of study population selection, four cohort studies [32, 33,, 36, 41] had dementia at baseline selection, three case-control studies [49, 51, 52] did not specify the definition of the control group, and one case-control study [52] did not explicitly describe the selection of the control population. In terms of comparability, six cohort studies [28, 32, 36–38, 44] and five case-control studies [45, 48, 49, 51, 52] were at risk of bias due to incomplete adjustment for confounding factors. Regarding outcomes, two studies [42, 43] were at risk of bias due to insufficient follow-up time. The mean quality score of the 27 studies was 7.59, indicating overall high methodological quality (Table 3).

**Table 2:** Methodological quality assessment results of the included cohort studies

Author, year	NOS selection domain				NOS comparability domain	NOS outcome domain			Total scores
	Representativeness of the exposed cohort	Selection of the non-exposed cohort	Ascertainment of exposure	Outcom not present at start	Comparability of cohorts on the basis of the design or analysis	Assessment of outcome	follow-uplong enough for outcomes to occur (median ≥ 5 years)	Adequacy of follow-up of cohorts	
Crump et al. 2024	☆	☆	☆	☆	☆☆	☆	☆	☆	9
Kim et al. 2023	☆	☆	☆	☆	☆	☆	☆	☆	8
Park et al. 2023	☆	☆	☆	☆	☆☆	☆	☆		8
Shang et al. 2023	☆	☆	☆	☆	☆☆	☆	☆	☆	9
Ekström et al. 2021	☆	☆	☆	☆	☆☆	☆	☆	☆	9
Belamkar et al.2021	☆	☆	☆		☆	☆	☆	☆	7
Hwang et al. 2021	☆	☆	☆		☆☆	☆	☆	☆	8
Su et al. 2016	☆	☆	☆	☆	☆☆	☆	☆		8
Chen et al. 2018	☆	☆	☆	☆	☆☆	☆		☆	8
Moon et al.2018	☆	☆	☆	☆	☆☆	☆	☆	☆	9
Xiao et al. 2020	☆	☆		☆	☆☆	☆	☆	☆	8
Keenan et al. 2015	☆	☆	☆		☆	☆	☆		6
Lee et al. 2018	☆	☆	☆	☆	☆	☆	☆		7
Ou et al. 2012	☆	☆	☆	☆	☆	☆	☆	☆	8
Lin et al. 2014	☆	☆	☆	☆	☆☆	☆	☆	☆	9
Kuo et al. 2020	☆	☆	☆	☆	☆☆	☆	☆	☆	9
Helmer et al. 2013	☆	☆	☆	☆	☆☆	☆		☆	8
Kessing et al. 2007	☆	☆	☆	☆	☆☆	☆			7
Ekstrom and Kilander 2016	☆	☆	☆		☆☆	☆	☆		7
Ekström and Kilander 2014	☆	☆	☆	☆	☆	☆	☆	☆	8

**Table 3:** Methodological quality assessment results of the included case-control studies

Author, year	Selection				Comparability	Exposure			Total scores
	Case definition and diagnosis	Representativeness of cases	Selection of control	Definition of control	Comparability of case and control	Identification of exposure factors	Investigation methods of case and control	Non-response rate	
Umunakwe et al. 2020	☆	☆			☆	☆	☆		5
Chamard et al. 2023	☆	☆	☆	☆	☆		☆		6
Chung et al. 2015	☆	☆	☆	☆	☆☆	☆	☆		8
Lai et al. 2017	☆	☆	☆	☆	☆	☆	☆		7
Wändell et al. 2022	☆	☆	☆		☆	☆	☆		6
Wang et al. 2021	☆	☆	☆	☆	☆		☆		6
Mullany et al. 2022	☆	☆	☆		☆	☆	☆		6

### Association between glaucoma and risk of all-cause dementia

**Fig. 2:**
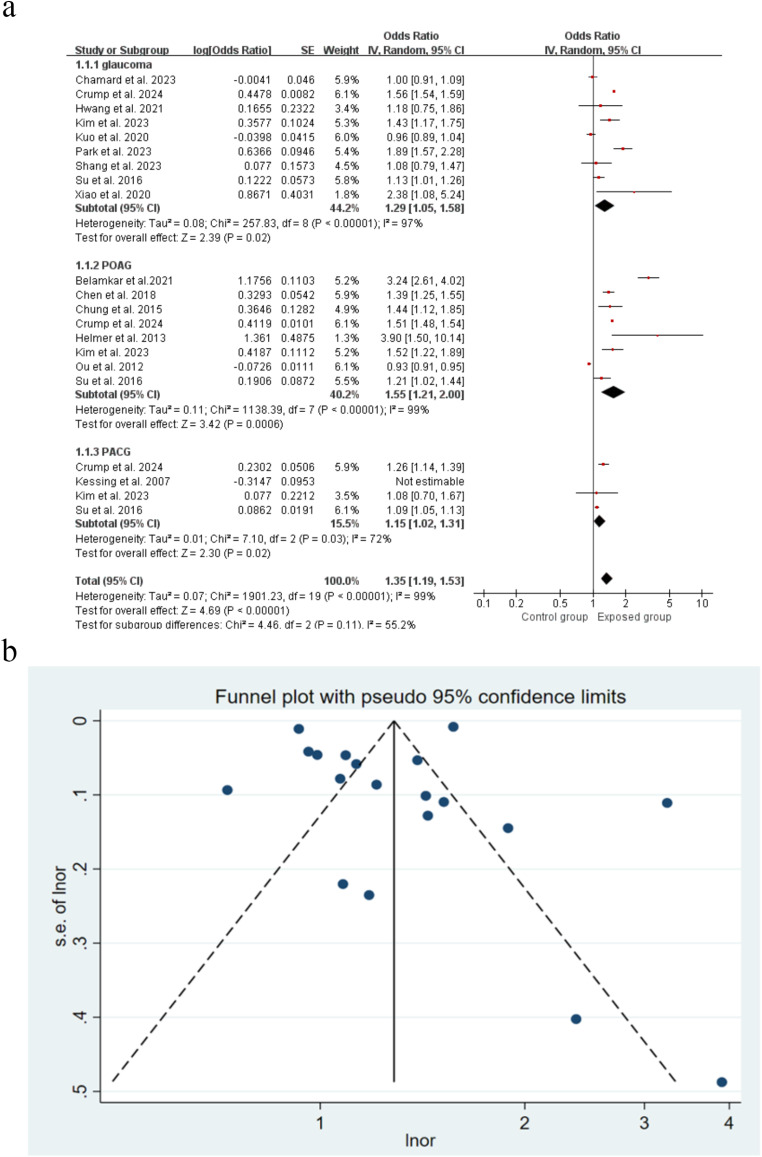
Forest plot and Funnel plot showing the effect of glaucoma on all-cause dementia

A total of 18 studies evaluated all-cause dementia as an outcome. Of these, 11 studies showed that glaucoma was associated with an increased risk of all-cause dementia, with effect estimates (OR) ranging from 1.09 (95% CI: 1.15˗1.13) to 3.90 (95% CI: 1.50˗10.14). In general, glaucoma was associated with an increased risk of all-cause dementia (OR = 1.31, 95% CI: 1.16˗1.48, *P* < 0.0001) (Fig. 2). Sensitivity analysis was performed by the trim-and-fill method, and the combined random-effects model resulted in a log OR = 0.259, 95% CI: 0.125˗0.393. After two iterations with the Linear method, the shear-complement method did not add to the study, indicating that the overall results were relatively robust (Supplementary Figure S1). Plotting funnel plots to test for publication bias showed that the distribution of studies was largely symmetrical (Fig. 2). Combining Egger’s test (*P* = 0.710) suggested a low likelihood of publication bias.

### Association between glaucoma and risk of Alzheimer’s disease

**Fig. 3:**
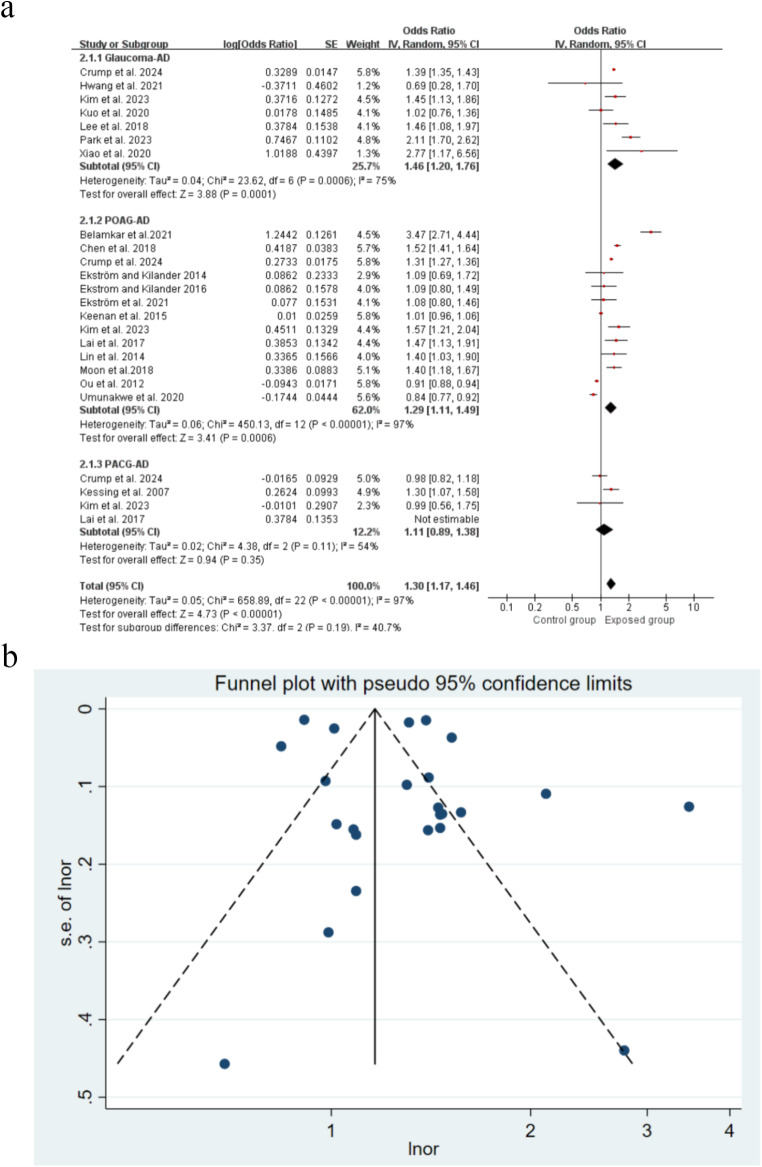
Forest plot and Funnel plot showing the effect of glaucoma on Alzheimer’s disease

There were 24 studies included, and the results revealed a strong link between glaucoma and an increased risk of Alzheimer’s disease (OR = 1.30, 95% CI: 1.17˗1.46, *P*<0.00001) (Fig. 3). For sensitivity analysis, using a study-by-study exclusion approach, the combined ORs ranged from 1.29 (1.13˗1.47) to 1.35 (1.15˗1.59) (Supplementary Figure S2). The funnel plot was roughly symmetrical (Fig. 3), and Egger’s test (*P* = 0.07) indicated that publication bias was unlikely.

### Association between glaucoma and risk of vascular dementia

**Fig. 4:**
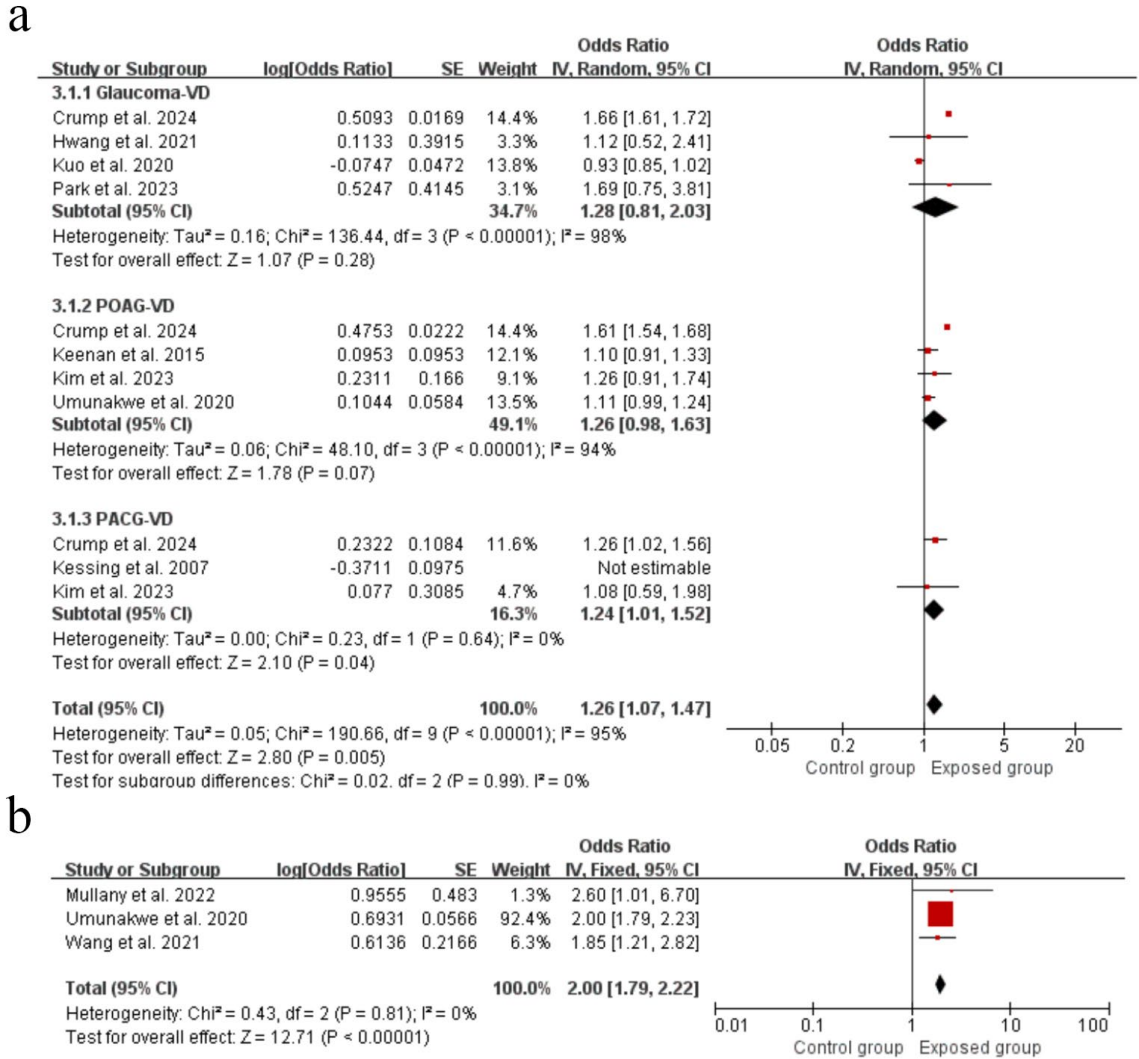
Forest plots showing the effect of glaucoma on vascular dementia (**a**) and cognitive impairment (**b**)

There was significant link between glaucoma and the risk of vascular dementia (OR = 1.26, 95% CI: 1.07˗1.47, *P* = 0.005) (Fig. 4). Removing one study at a time did not have a statistically significant effect on the size of the aggregated results in the sensitivity analysis (Supplementary Figure S3). Regarding publication bias, Egger’s test (*P* = 0.436) also suggested no publication bias .

### Association between glaucoma and risk of cognitive impairment

Three studies with a total of 1,513,047 patients were included, and the result revealed that glaucoma patients had twice the risk of cognitive impairment as the general population (OR = 2.00, 95% CI: 1.79˗2.22, *P*<0.00001) (Fig. 4). Egger’s test (*P* = 0.800) indicated that there was no significant publication bias.

### Subgroup analysis

Subgroup analysis revealed that the type of glaucoma, gender, age and region of the study population significantly influenced the relationship between glaucoma and dementia. The pooled results showed that primary open-angle glaucoma increased the risk of all-cause dementia (OR = 1.55, 95% CI: 1.21˗2.00, *P* = 0.0006), Alzheimer’s disease (OR = 1.29, 95% CI: 1.11˗1.49, *P* = 0.0006), and cognitive impairment (OR = 2.00, 95% CI: 1.79˗2.22, *P*<0.0001), while angle-closure glaucoma increased the risk of vascular dementia (OR = 1.24, 95% CI: 1.01˗1.52, *P* = 0.04). Concerning gender, female glaucoma patients were more likely to develop Alzheimer’s disease (OR = 1.82, 95% CI: 1.47˗2.26, *P*<0.00001), whereas there was no significant link between male glaucoma patients and dementia. According to age subgroup analyses, glaucoma patients aged ≥ 65 or < 65 both had a significantly increased risk of all-cause dementia and Alzheimer’s disease. In addition, we found that the glaucoma patients in Asia had a 29% increased risk of all-cause dementia, and a 48% increased risk of Alzheimer’s disease compared to those without the visual condition. In Europe and North America, nevertheless, there was no correlation between glaucoma and dementia. The results from subgroup analyses by sample size and follow-up time showed no statistically significant differences regarding the impact of glaucoma on dementia in the subgroups, and they were not the source of heterogeneity (Table 4).

**Table 4:** Subgroup analyses of the influence of glaucoma on dementia

Variable		Number of studies	Heterogeneity	Effect model	Mate-analysis	
					Pooled OR (95% CI)	*P*-value
(**Types of dementia**)						
All-cause dementia		18	*P*<0.00001; I^2^ = 99%	random-effects model	1.35(1.19, 1.53)	<0.00001
Alzheimer’s disease		24	*P*<0.00001; I^2^ = 97%	random-effects model	1.30(1.17, 1.46)	<0.00001
Vascular dementia		11	*P*<0.00001; I^2^ = 95%	random-effects model	1.26(1.07, 1.47)	0.005
**Types of glaucoma**						
POAG	All-cause dementia	8	*P*<0.00001; I^2^ = 99%	random-effects model	1.55(1.21, 2.00)	0.0006
	Alzheimer’s disease	13	*P*<0.00001; I^2^ = 97%	random-effects model	1.29(1.11, 1.49)	0.0006
	Vascular dementia	4	*P*<0.00001; I^2^ = 94%	random-effects model	1.26(0.98, 1.63)	0.07
PACG	All-cause dementia	4	*P*<0,001; I^2^ = 88%	random-effects model	1.03(0.86, 1.48)	0.74
	Alzheimer’s disease	3	*P* = 0.11; I^2^ = 54%	random-effects model	1.11(0.89, 1.38)	0.35
	Vascular dementia	3	*P* = 0.64; I^2^ = 0%	fixed-effects model	1.24(1.01, 1.52)	0.04
**Sex**						
Man	Overall	11	*P*<0.00001; I^2^ = 93%	random-effects model	1.07(0.97, 1.19)	0.17
	All-cause dementia	6	*P*<0.00001; I^2^ = 95%	random-effects model	1.04(0.84, 1.27)	0.74
	Alzheimer’s disease	5	*P*<0.00001; I^2^ = 89%	random-effects model	1.21(0.94, 1.57)	0.14
Woman	Overall	10	*P*<0.00001; I^2^ = 98%	random-effects model	1.46(1.06, 2.00)	0.02
	All-cause dementia	7	*P*<0.00001; I^2^ = 98%	random-effects model	1.34(0.91, 1.98)	0.14
	Alzheimer’s disease	3	*P* = 0.37; I^2^ = 0%	fixed-effects model	1.82(1.47, 2.26)	<0.00001
**Age**						
≥ 65 years	Overall	7	*P*<0.00001; I^2^ = 99%	random-effects model	2.90(1.45, 5.77)	0.003
	All-cause dementia	3	*P*<0.00001; I^2^ = 93%	random-effects model	1.26(1.13, 1.41)	<0.0001
	Alzheimer’s disease	4	*P*<0.00001; I^2^ = 98%	random-effects model	4.53(1.08, 19.04)	0.04
<65 years	Overall	6	*P*<0.00001; I^2^ = 95%	random-effects model	2.07(1.18, 3.62)	0.01
	All-cause dementia	3	*P* = 0.74; I^2^ = 0%	fixed-effects model	1.49(1.25, 1.77)	<0.00001
	Alzheimer’s disease	3	*P*<0.00001; I^2^ = 97%	random-effects model	3.01(1.11, 8.85)	0.03
**Sample size**						
≥ 10,000	All-cause dementia	12	*P*<0.00001; I^2^ = 99%	random-effects model	1.16(0.97, 1.39)	0.10
	Alzheimer’s disease	9	*P*<0.00001; I^2^ = 99%	random-effects model	1.22(1.02, 1.45)	0.03
	Vascular dementia	6	*P*<0.00001; I^2^ = 98%	random-effects model	1.10(0.80, 1.52)	0.55
<10,000	All-cause dementia	7	*P*<0.00001; I^2^ = 87%	random-effects model	1.78(1.24, 2.54)	0.002
	Alzheimer’s disease	12	*P*<0.00001; I^2^ = 82%	random-effects model	1.43(1.15, 1.79)	0.001
	Vascular dementia	3	*P =* 0.89; I^2^ = 0%	fixed-effects model	1.20(0.92, 1.58)	0.17
**Mean follow-up time**						
≥ 10 years	All-cause dementia	8	*P*<0.00001; I^2^ = 97%	random-effects model	1.28(1.11, 1.48)	0.0008
	Alzheimer’s disease	11	*P*<0.00001; I^2^ = 95%	random-effects model	1.35(1.13, 1.60)	0.0007
	Vascular dementia	3	*P =* 0.11; I^2^ = 55%	random-effects model	1.02(0.85, 1.22)	0.84
<10 years	All-cause dementia	9	*P*<0.00001; I^2^ = 84%	random-effects model	1.33(1.07, 1.66)	0.01
	Alzheimer’s disease	10	*P*<0.00001; I^2^ = 92%	random-effects model	1.31(1.04, 1.65)	0.02
	Vascular dementia	5	*P =* 0.0006; I^2^ = 80%	random-effects model	1.00(0.76, 1.33)	0.99
**Geographic location**						
Asia	All-cause dementia	11	*P =* 0.0006; I^2^ = 88%	random-effects model	1.29(1.15, 1.45)	<0.0001
	Alzheimer’s disease	11	*P =* 0.02; I^2^ = 54%	random-effects model	1.48(1.33, 1.65)	<0.00001
	Vascular dementia	4	*P =* 0.16; I^2^ = 42%	fixed-effects model	0.96(0.88, 1.05)	0.34
Europe	All-cause dementia	5	*P*<0.00001; I^2^ = 96%	random-effects model	1.18(0.83, 1.68)	0.34
	Alzheimer’s disease	6	*P*<0.00001; I^2^ = 96%	random-effects model	1.17(0.96, 1.41)	0.12
	Vascular dementia	3	*P*<0.00001; I^2^ = 98%	random-effects model	1.09(0.63, 1.88)	0.76
North America	All-cause dementia	3	*P*<0.00001; I^2^ = 98%	random-effects model	0.94(0.92, 0.96)	<0.00001
	Alzheimer’s disease	5	*P*<0.00001; I^2^ = 97%	random-effects model	1.28(0.93, 1.78)	0.13
	Vascular dementia	2	*P =* 0.98; I^2^ = 0%	fixed-effects model	1.11(0.99, 1.24)	0.07
**MCI**						
POAG		3	*P =* 0.81; I^2^ = 0%	fixed-effects model	2.00(1.79, 2.22)	<0.00001

OR, odds ratio; CI, confidence interval; NA, not applicable

*P < 0.05 for the Q-test or I^2^ > 50% indicated significant heterogeneity

## Discussion

### Main findings

The present study comprehensively investigated the association between glaucoma and the risk of incident dementia or cognitive decline and found that glaucoma was an independent risk factor for all-cause dementia, Alzheimer’s disease, vascular dementia and cognitive impairment. Our result is similar to the findings of a recently published systematic review. Xu et al. [53] quantified the association between glaucoma and cognitive impairment or dementia, proposing a prevalence of 7.7% for glaucoma patients with mild cognitive impairment; 3.9–77.8% for cognitive impairment and 2.5–3.3% for dementia in glaucoma patients.

Several meta-analyses have tried to pool the effects of the associations of glaucoma with dementia or cognitive decline. The meta-analysis by Zhao et al. [54]. included only eleven cohort studies published up to 2020 and revealed that glaucoma was not an independent risk factor for dementia. Similarly, Kuźma et al. [55] performed a meta-analysis of eight cross-sectional studies involving 175,357 individuals up to 2020 and found no association between glaucoma and dementia. On the contrary, our systematic review and meta-analysis is the comprehensive meta-analysis to confirm that glaucoma is linked to dementia, which may contribute to an accurate assessment of whether glaucoma patients are associated with an increased risk of dementia or cognitive impairment.

Notwithstanding these overall associations, there was a high heterogeneity of effects across all the studies and sensitivity analyses did not reduce the heterogeneity. Our subgroup analysis showed that glaucoma type, gender, age, and region (ethnicity) were influential factors in the association between glaucoma and the risk of dementia. In addition, the different confounders adjusted for each study may be another source of heterogeneity. However, there was a nonsignificant association between glaucoma and all-cause dementia in subgroups with follow-up years > 10, age 65and number of cases ≥ 10,000.

Angle-closure glaucoma only increases the occurrence of vascular dementia. Vascular imaging has shown evidence of microvascular dysfunction in both angle-closure glaucoma and dementia [56]. According to Cruz Hernández et al. [57], age-related decreased angiogenesis, lessened vascular vessel diameter, inefficient cell signaling, and impaired vasodilation result in reduced cerebral blood flow. Intermittent cerebral ischemia is linked to vascular dementia, and ischemia can cause oxidative stress, leading to the formation of reactive oxygen species and cellular damage [58]. The accumulation of neurotoxic factors causes cell death in Alzheimer’s disease, and it has been linked to retinal ganglion cell death in glaucoma. PACG patients have microvascular dysfunction and deficiencies in endothelial-dependent and non-endothelial-dependent vasodilatory responses [59, 60]. However, open-angle glaucoma was not associated with vascular dementia, possibly due to open-angle glaucoma being included in fewer observational studies, reducing statistical efficiency. More studies are needed to confirm the association between open-angle glaucoma and dementia.

The results of subgroup analyses revealed gender differences in the prevalence of dementia, with women with a higher risk of Alzheimer’s disease with glaucoma, which is consistent with epidemiological research on dementia. Furthermore, clinical evidence has shown that women have faster age-related neurological decline and more severe cognitive impairment than elderly men [61]. Currently, there are several major biological hypotheses regarding gender differences in Alzheimer’s disease, including age-related decreases in sex hormones (estrogen, progesterone, testosterone), various genetic risks (ApoE, Etc.), effects of risk for other diseases (diabetes, depression, cardiovascular disease), and gender differences in brain anatomy [62, 63]. Estradiol, for example, increases neurogenesis in different brain regions, such as the hippocampal dentate gyrus, and these newly generated neurons in the hippocampus contribute to region-specific learning and memory [64]. Women with mild cognitive impairment (MCI) had hippocampal atrophy, confirming estrogen’s critical role in cognitive function [65]. However, there is no link between brain estrogen levels and the onset of dementia in men [66], which may explain our results that there was no significant association between male glaucoma patients and the risk of dementia.

Age subgroup analysis proved that the coexistence of glaucoma and dementia became more pronounced with age. The risk of dementia was 2.9 times higher in glaucoma patients aged 65 years, and Alzheimer’s disease was 4.09 times higher, presumably due to the increased prevalence of glaucoma and dementia associated with advancing age. Furthermore, we discovered that the risk of glaucoma and all-cause dementia or Alzheimer’s disease differed geographically. In Asia, glaucoma significantly increased the risk of all-cause dementia and Alzheimer’s disease, whereas there was no correlation in Europe or North America. We speculate that it may be related to the large population base, accelerated population aging, and increased level of dementia diagnosis and dementia reporting in Asia, resulting in an overall high prevalence of dementia associated with rapid growth [67].

### Strengths and limitations

Our study encompassed data from 27 population-based longitudinal studies, including 9,061,675 participants across 8 countries, with considerable sample size. Compared with previous meta-analyses, we attempted to include all forms of dementia by including all relevant studies, not only subgroup analysis of the type of glaucoma, the subtype of dementia, age, gender, sample size, geographic location, and follow-up time, but also a refined analysis of the association of each influencing factor with the subtype of dementia.

Nevertheless, the study still had a few potential limitations. First, there was considerable heterogeneity among the results of the included studies, lowering the quality of the evidence. Based on our findings, the type of glaucoma and geographic region of the study population may be a source of heterogeneity. Alternatively, different approaches to assessing glaucoma, dementia, and the variables used to characterize cognitive decline may provide other explanations for the discrepancy. Nevertheless, due to the limitations of the original data, more detailed subgroup analyses could not be performed, and the available results did not fully explain the sources of heterogeneity generation. None of the studies recruited participants immediately at the time of glaucoma diagnosis, which may have led to selection bias. Second, some did not fully adjust for confounding factors, such as BMI, smoking, and alcohol consumption, all risk factors for dementia or cognitive impairment. These factors may influence the association between glaucoma and dementia or cognitive impairment. Finally, the study subjects were from different regions, and differences between races may have impacted the results; the included studies were all cohort studies and case-control studies, with a higher risk of various types of bias, such as selection and recall bias.

Despite these limitations, our study has implications for public health, government officials, researchers, and the general public. The global population is growing and advances in health care and social welfare have prolonged life expectancy, meaning that older adults will represent a significant proportion of the population. As a result, age-related diseases such as Alzheimer’s disease will become more prevalent. More high-quality longitudinal studies are needed in the future to assess the association between glaucoma subtypes and dementia risk and to identify sources of heterogeneity in previously published studies. At the same time, the factors influencing the relationship between glaucoma and dementia or cognitive function should be further explored and fully adjusted to identify the underlying biological basis and reveal features of glaucoma that may increase the risk of dementia, and translational studies as well as clinical and population studies are necessary to determine the impact of different treatment strategies and the degree of glaucoma disease on cognitive function, and ultimately to identify targeted preventive interventions.

## Conclusions

Overall, our review first demonstrated that glaucoma increased the risk of subsequent cognitive impairment and dementia, including all-cause dementia, Alzheimer’s disease, and vascular dementia, which updated the results of previous meta-analysis. These findings contribute to the further promotion of dementia awareness and glaucoma patients, and serve to develop global management strategies to reduce the occurrence of dementia in glaucoma.

### Electronic supplementary material

Below is the link to the electronic supplementary material.

Supplementary Material 1

Supplementary Material 2

Supplementary Material 3

Supplementary Material 4

Supplementary Material 5

Supplementary Material 6

## Data Availability

No datasets were generated or analysed during the current study.
