# Supplementary material for: Risk of glaucoma to subsequent dementia or cognitive impairment: a systematic review and meta-analysis
Source: Aging Clin Exp Res. 2024 Aug 20;36(1):172. doi: 10.1007/s40520-024-02811-w (PMC11335947; doi:10.1007/s40520-024-02811-w)
Supplement: Supplementary file 2 — Supplementary Material 2 [file 40520_2024_2811_MOESM2_ESM.pdf]

Supplementary Appendix 2

Details of the Literature Search Strategy

(1) PubMed

No.	Content	Result
#1	"glaucoma"[MeSH Terms]	59,046
#2	"glaucoma"[Title/Abstract]	68,392
#3	#1 OR #2	80083
#4	"dementia"[MeSH Terms]	200,453
#5	"dementia"[Title/Abstract] OR "Alzheimer's disease"[Title/Abstract] OR "vascular dementia"[Title/Abstract] OR "senile dementia"[Title/Abstract] OR "cognitive decline"[Title/Abstract] OR "cognitive disorder"[Title/Abstract] OR "cognitive dysfunction"[Title/Abstract] OR "cognitive impairment"[Title/Abstract]	312377
#6	#4 OR #5	361,819
#7	#3 AND #6	631

Table 2: Cochrane Library

No.	Content	Result
#1	MeSH descriptor: [Glaucoma] explode all trees	3740
#2	(Glaucoma):ti,ab,kw	8624
#3	#1 or #2	8624
#4	MeSH descriptor: [Dementia] explode all trees	7943
#5	(dementia):ti,ab,kw OR (Alzheimer's disease):ti,ab,kw OR (vascular dementia):ti,ab,kw OR (senile dementia):ti,ab,kw OR (cognitive decline):ti,ab,kw OR (cognitive impairment):ti,ab,kw OR (cognitive	55352

	disorder):ti,ab,kw OR (cognitive dysfunction):ti,ab,kw	
#6	#4 or #5	55633
#7	#3 and #6	49

Table 3: Web of Science

No.	Content	Result
#1	TS=(Glaucoma)	108,398
#2	(((((((TS=(dementia)) OR TS=(Alzheimer’s disease)) OR TS=(cognitive decline)) OR TS=(cognitive impairment)) OR TS=(cognitive disorder)) OR TS=(cognitive dysfunction)) OR TS=(vascular dementia)) OR TS=(Senile dementia)	709,062
#3	#1 AND #2	1249

Table 4: Embase

No.	Content	Result
#1	'glaucoma'/exp OR glaucoma	123,254
#2	'dementia'/exp OR 'dementia' OR 'alzheimer disease'/exp OR 'alzheimer disease' OR 'vascular dementia'/exp OR 'vascular dementia' OR 'senile dementia'/exp OR 'senile dementia' OR 'cognitive decline'/exp OR 'cognitive decline' OR 'cognitive impairment'/exp OR 'cognitive impairment' OR 'cognitive disorder'/exp OR 'cognitive disorder' OR 'cognitive dysfunction'/exp OR 'cognitive dysfunction'	660,821
#3	#1 and #2	2287
