# Supplementary material for: Risk of glaucoma to subsequent dementia or cognitive impairment: a systematic review and meta-analysis
Source: Aging Clin Exp Res. 2024 Aug 20;36(1):172. doi: 10.1007/s40520-024-02811-w (PMC11335947; doi:10.1007/s40520-024-02811-w)
Supplement: Supplementary file 3 — Supplementary Material 3 [file 40520_2024_2811_MOESM3_ESM.pdf]

Supplementary Appendix 3

Excluded studies after reading the full text

Author, Year	Tittle	Reasons
Taloyan, M, 2023	Physical-mental multimorbidity in a large primary health care population in Stockholm County, Sweden	Not meet the inclusion criteria for participants
Wang, X, 2022	The macular inner plexiform layer thickness as an early diagnostic indicator for Parkinson's disease	
Bertsias, A. K, 2018	Cognitive impairment in a primary healthcare population: A cross-sectional study on the island of Crete, Greece	
Davies-Kershaw R, 2018	Vision Impairment and Risk of Dementia: Findings from the English Longitudinal Study of Ageing	
Yang, Y, 2018	Ten-year cumulative incidence of epiretinal membranes assessed on fundus photographs. The Beijing eye study 2001/ 2011	
Jonas, J. B, 2018	Cognitive Function and Ophthalmological Diseases: The Beijing Eye Study	
Chriqui, E, 2017	Visual impairment in older institutionalised Canadian seniors with dementia	
Maurano, S. T. P, 2018	Cognitive evaluation of patients with glaucoma and its comparison with individuals with Alzheimer’s disease	

Yoshikawa, T, 2021	Lower Cognitive Function in Patients With Functionally and Structurally Severe Glaucoma: The LIGHT Study	
Wostyn, P, 2017	Alzheimer’s disease and glaucoma: can glymphatic system dysfunction underlie their comorbidity?	The type of study does not match
Vidal, K. S, 2020	Association between cognitive performance and self-reported glaucoma in middle-aged and older adults: a cross-sectional analysis of ELSA-Brasil	
Norouzpour, A, 2021	Glaucomatous Optic Neuropathy: Associated With Cognitive Impairment?	Full text of the study is not available
Mutlu, U, 2018	Association of Retinal Neurodegeneration on Optical Coherence Tomography with Dementia: A Population-Based Study	
Varin, M, 2017	Age-Related Eye Disease and Participation in Cognitive Activities	
Honjo, M, 2017	The association between structure-function relationships and cognitive impairment in elderly glaucoma patients	
McCoskey, M, 2018	Association between cognitive impairment and primary open-angle glaucoma using the montreal cognitive assessment	
Daveckaite, A, 2017	Cognitive functions and normal tension glaucoma	

Bulut, M, 2016	Cognitive performance of primary open-angle glaucoma and normal-tension glaucoma patients	Without relevant outcomes
Pelletier, A. A, 2014	Prevalence of glaucoma in hospitalized older adults with alzheimer's disease	
Jefferis, J. M, 2013	The association between diagnosed glaucoma and cataract and cognitive performance in very old people: Cross-sectional findings from the Newcastle 85+ Study	
Varin, M, 2020	Age-Related Eye Disease and Cognitive Function: The Search for 2 Mediators	
Varin, M, 2017	Age-Related Eye Disease and Participation in Cognitive Activities	
Sahoo, S, 2018	Association of Well-Being Index and Cognitive Impairment with Primary Open Angle Glaucoma Patients of Malaysia: A Case-Control Study	
Honjo, M, 2017	The association between structure function relationships and cognitive impairment in elderly glaucoma patients	
Raman, P, 2019	The Association Between Visual Field Reliability Indices and Cognitive Impairment in Glaucoma Patients	
McCoskey, M, 2018	Association between Primary Open-Angle Glaucoma and Cognitive Impairment As Measured by the Montreal Cognitive Assessment	
