# Supplementary material for: Risk of glaucoma to subsequent dementia or cognitive impairment: a systematic review and meta-analysis
Source: Aging Clin Exp Res. 2024 Aug 20;36(1):172. doi: 10.1007/s40520-024-02811-w (PMC11335947; doi:10.1007/s40520-024-02811-w)
Supplement: Supplementary file 4 — Supplementary Material 4 [file 40520_2024_2811_MOESM4_ESM.pdf]

Supplementary Appendix 4

Sensitivity Analysis

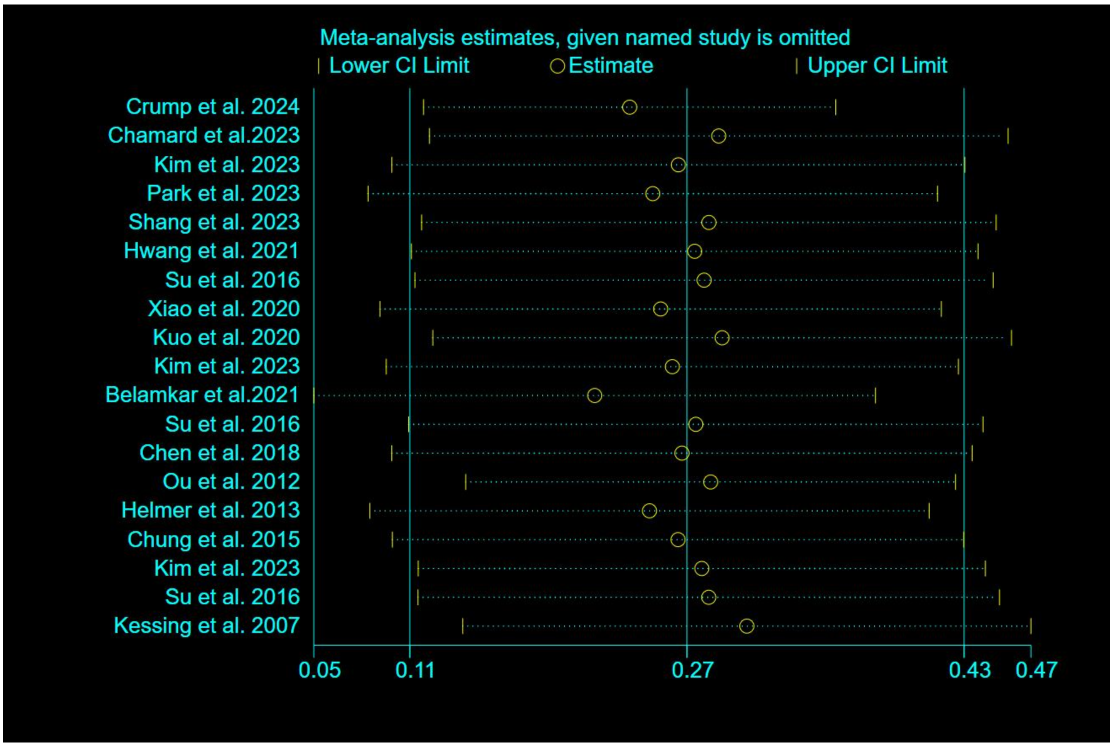

Trim-and-fill method

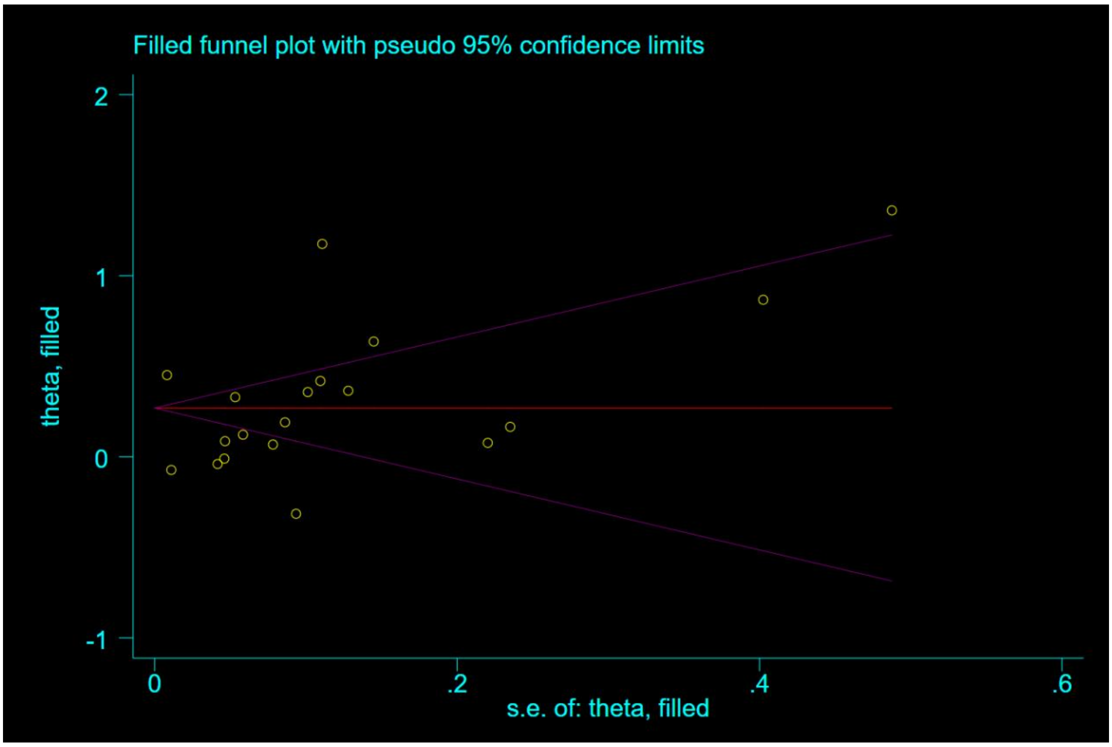

Figure S1 –Sensitivity analysis showing the effect of glaucoma on all-cause dementia

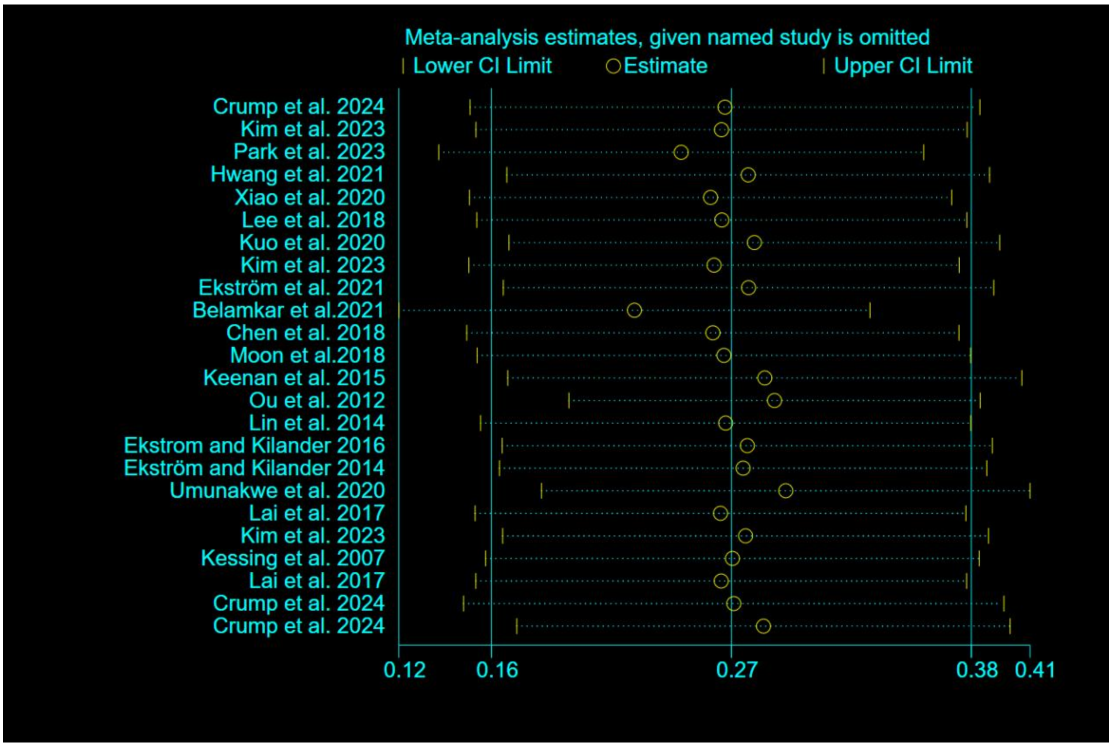

Trim-and-fill method

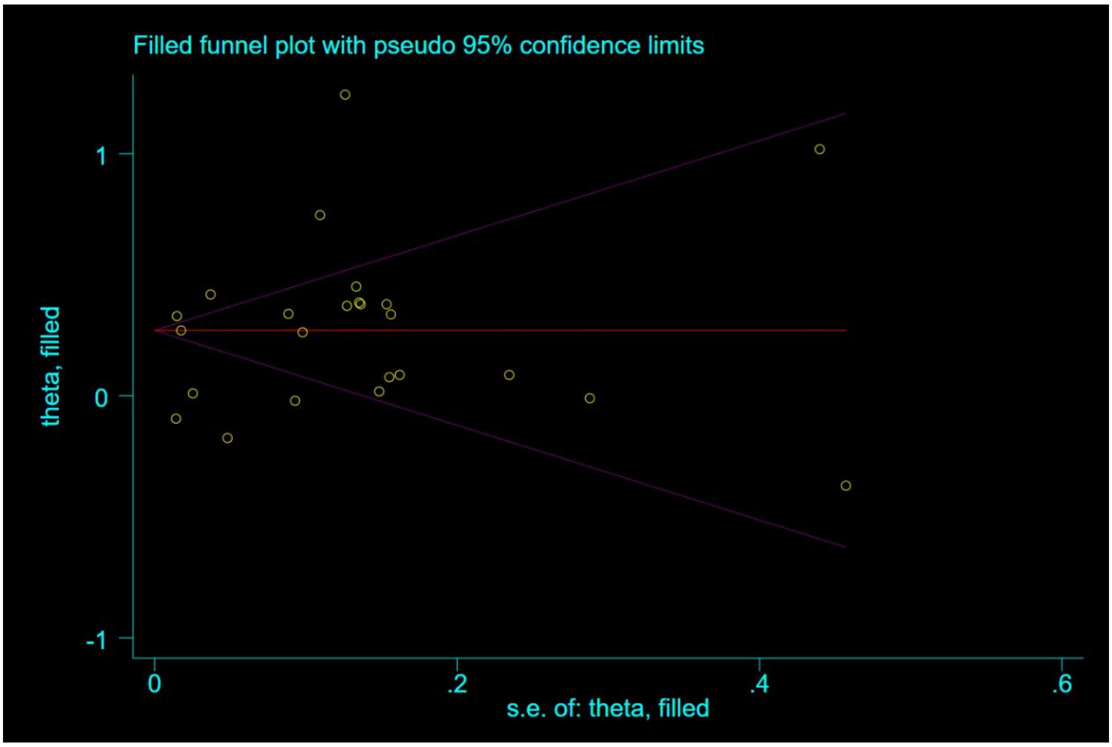

Figure S2–Sensitivity analysis showing the effect of glaucoma on Alzheimer's disease

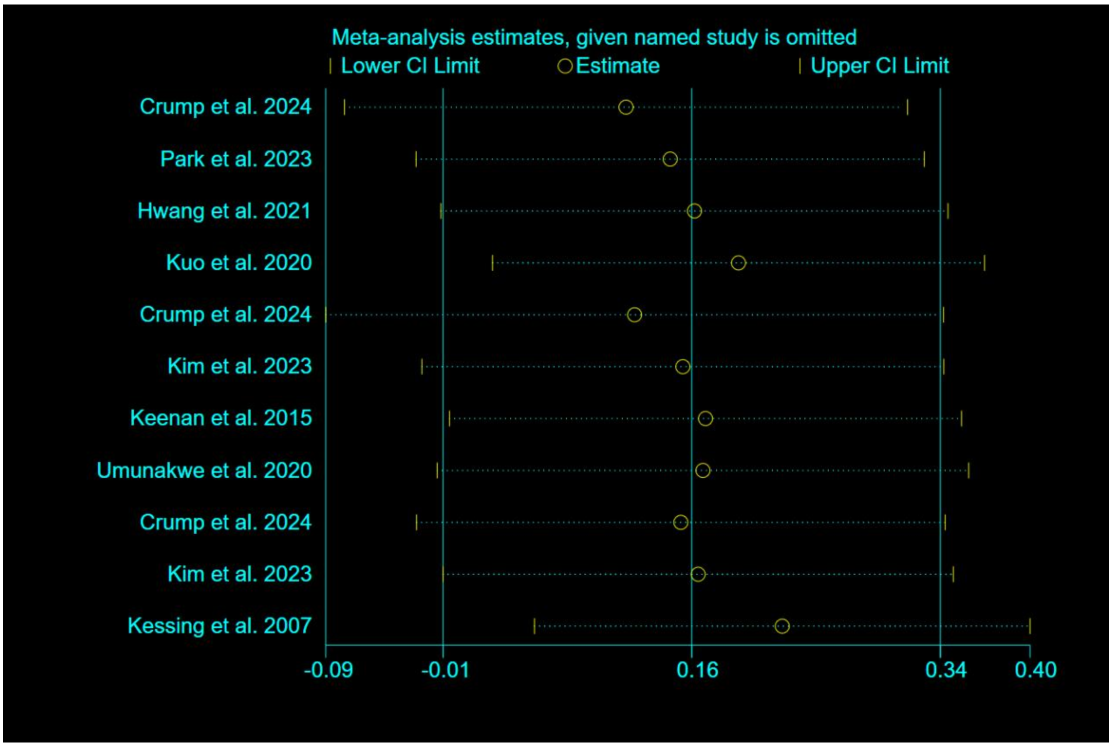

Trim-and-fill method

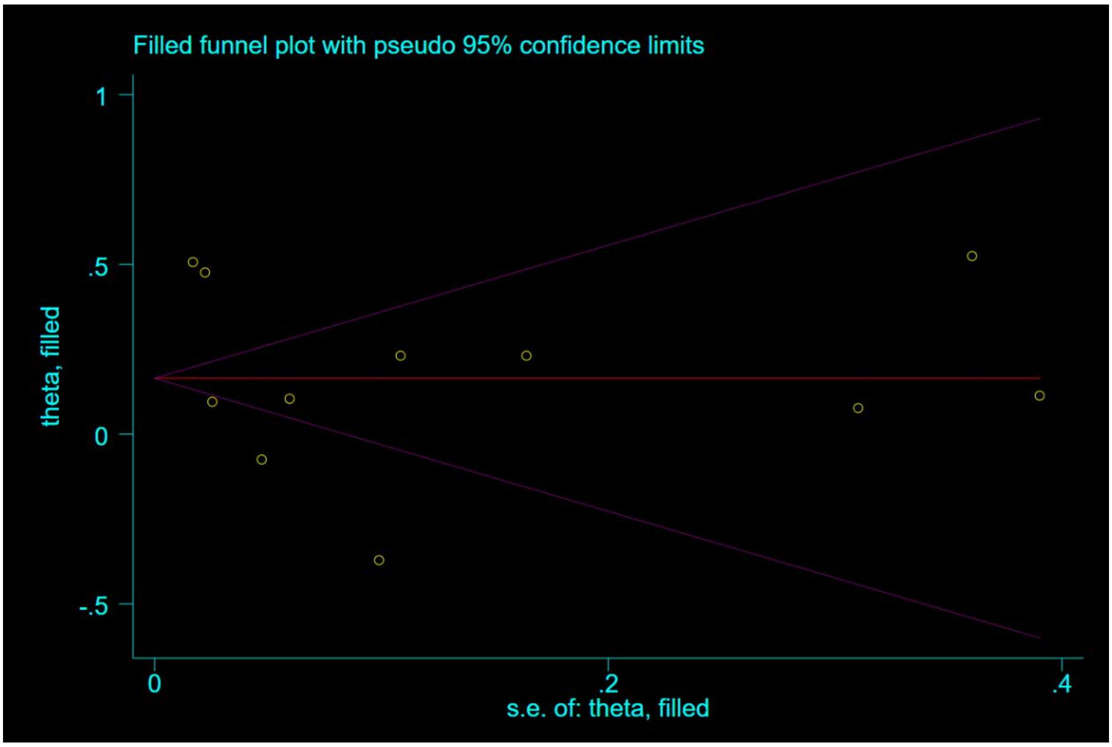

Figure S3 –Sensitivity analysis showing the effect of glaucoma on vascular dementia

Table S1 –Sensitivity analysis showing the effect of glaucoma on all-cause dementia

Deletion	Result
Crump et al. 2024	OR=1.30, 95% CI [1.14, 1.48]
Crump et al. 2024(POAG)	OR=1.31, 95% CI [1.13, 1.53]
Crump et al. 2024(PACG)	OR=1.31, 95% CI [1.16, 1.49]
Kim et al. 2023	OR=1.30, 95% CI [1.15, 1.48]
Park et al. 2023	OR=1.28, 95% CI [1.13, 1.46]
Shang et al. 2023	OR=1.32, 95% CI [1.16, 1.50]
Hwang et al. 2021	OR=1.31, 95% CI [1.16, 1.49]
Su et al. 2016	OR=1.32, 95% CI [1.16, 1.50]
Xiao et al. 2020	OR=1.30, 95% CI [1.14, 1.47]
Kuo et al. 2020	OR=1.33, 95% CI [1.18, 1.51]
Chamard et al. 2023	OR=1.33, 95% CI [1.17, 1.51]
Kim et al. 2023(POAG)	OR=1.30, 95% CI [1.15, 1.47]
Belamkar et al. 2021	OR=1.25, 95% CI [1.10, 1.41]
Su et al. 2016(POAG)	OR=1.32, 95% CI [1.16, 1.49]
Chen et al. 2018	OR=1.31, 95% CI [1.15, 1.48]
Ou et al. 2012	OR=1.32, 95% CI [1.21, 1.45]
Helmer et al. 2013	OR=1.29, 95% CI [1.14, 1.46]
Chung et al. 2015	OR=1.30, 95% CI [1.15, 1.48]
Kim et al. 2023(PACG)	OR=1.32, 95% CI [1.16, 1.49]
Su et al. 2016(PACG)	OR=1.33, 95% CI [1.16, 1.51]
Kessing et al. 2007	OR=1.35, 95% CI [1.19, 1.53]

Table S2 –Sensitivity analysis showing the effect of glaucoma on Alzheimer's disease

Deletion	Result
Crump et al. 2024	OR=1.31, 95% CI [1.16, 1.47]
Crump et al. 2024(POAG)	OR=1.31, 95% CI [1.16, 1.48]
Crump et al. 2024(PACG)	OR=1.33, 95% CI [1.19, 1.48]
Kim et al. 2023	OR=1.30, 95% CI [1.17, 1.46]
Park et al. 2023	OR=1.28, 95% CI [1.15, 1.43]
Hwang et al. 2021	OR=1.32, 95% CI [1.18, 1.47]
Xiao et al. 2020	OR=1.30, 95% CI [1.17, 1.45]
Lee et al. 2018	OR=1.30, 95% CI [1.17, 1.46]
Kuo et al. 2020	OR=1.32, 95% CI [1.19, 1.48]
Kim et al. 2023(POAG)	OR=1.30, 95% CI [1.16, 1.45]
Ekström et al. 2021	OR=1.32, 95% CI [1.18, 1.47]
Belamkar et al. 2021	OR=1.25, 95% CI [1.13, 1.39]
Chen et al. 2018	OR=1.30, 95% CI [1.16, 1.45]
Moon et al. 2018	OR=1.31, 95% CI [1.17, 1.46]
Keenan et al. 2015	OR=1.33, 95% CI [1.19, 1.49]
Ou et al. 2012	OR=1.34, 95% CI [1.21, 1.47]
Lin et al. 2014	OR=1.31, 95% CI [1.17, 1.46]
Ekstrom and Kilander 2016	OR=1.32, 95% CI [1.18, 1.47]
Ekstrom and Kilander 2014	OR=1.32, 95% CI [1.18, 1.47]
Umunakwe et al. 2020	OR=1.34, 95% CI [1.21, 1.50]
Lai et al. 2017(POAG)	OR=1.30, 95% CI [1.17, 1.46]
Kim et al. 2023(PACG)	OR=1.32, 95% CI [1.18, 1.47]
Kessing et al. 2007	OR=1.31, 95% CI [1.17, 1.46]
Lai et al. 2017(PACG)	OR=1.30, 95% CI [1.17, 1.46]

Table S3 –Sensitivity analysis showing the effect of glaucoma on vascular dementia

Deletion	Result
Crump et al. 2024	OR=1.36, 95% CI [1.31, 1.41]
Crump et al. 2024(POAG)	OR=1.47, 95% CI [1.43, 1.52]
Crump et al. 2024(PACG)	OR=1.52, 95% CI [1.48, 1.55]
Park et al. 2023	OR=1.51, 95% CI [1.48, 1.55]
Hwang et al. 2021	OR=1.51, 95% CI [1.48, 1.55]
Kuo et al. 2020	OR=1.57, 95% CI [1.53, 1.61]
Kim et al. 2023(POAG)	OR=1.52, 95% CI [1.48, 1.55]
Keenan et al. 2015	OR=1.52, 95% CI [1.49, 1.56]
Umunakwe et al. 2020	OR=1.54, 95% CI [1.50, 1.57]
Kin et al. 2023(PACG)	OR=1.51, 95% CI [1.48, 1.55]
Kessing et al. 2007	OR=1.53, 95% CI [1.50, 1.57]
