# Supplementary material for: Risk of glaucoma to subsequent dementia or cognitive impairment: a systematic review and meta-analysis
Source: Aging Clin Exp Res. 2024 Aug 20;36(1):172. doi: 10.1007/s40520-024-02811-w (PMC11335947; doi:10.1007/s40520-024-02811-w)
Supplement: Supplementary file 5 — Supplementary Material 5 [file 40520_2024_2811_MOESM5_ESM.pdf]

Supplementary Appendix 5

Subgroup analysis

①Gender

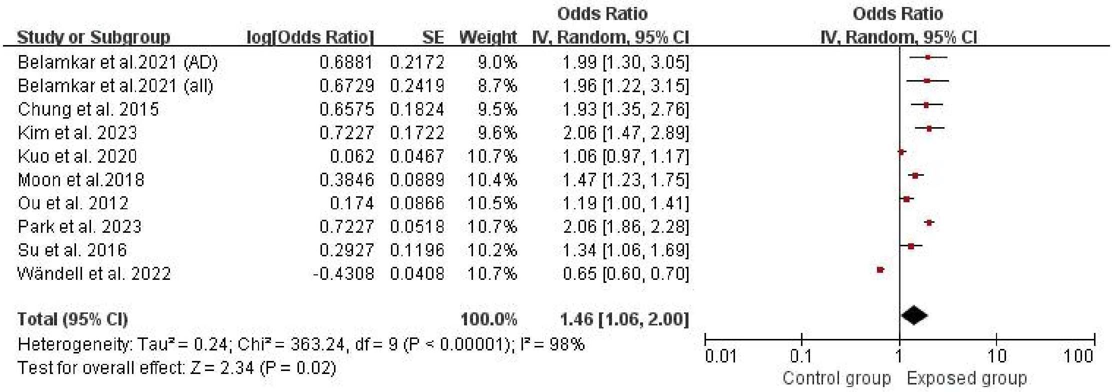

Figure S4. Forest plot showing the effect of female glaucoma patients on dementia

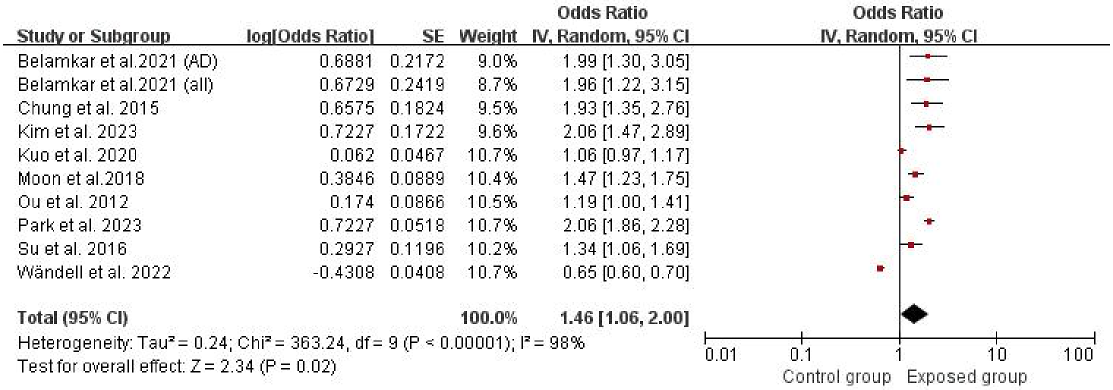

Figure S5. Forest plot showing the effect of male glaucoma patients on dementia

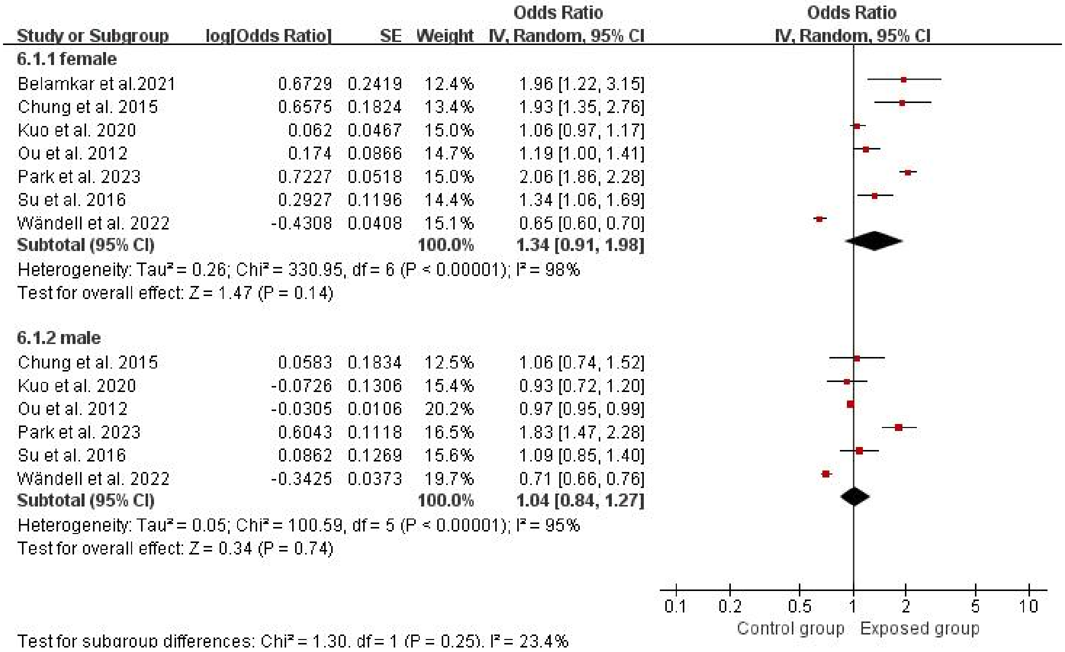

Figure S6. Forest plot showing the effect of gender on all-cause dementia with glaucoma

Random-effects model

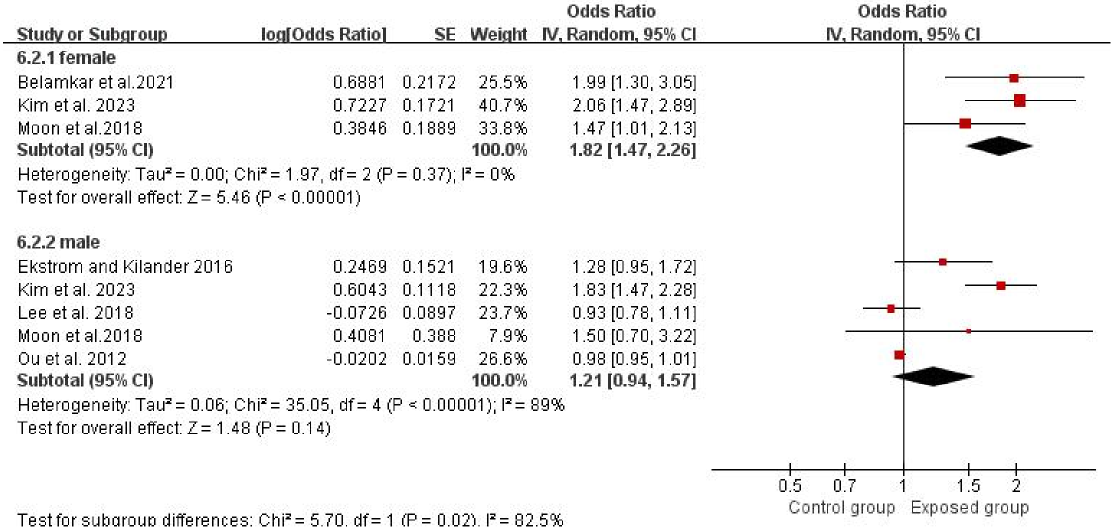

fixed-effects model

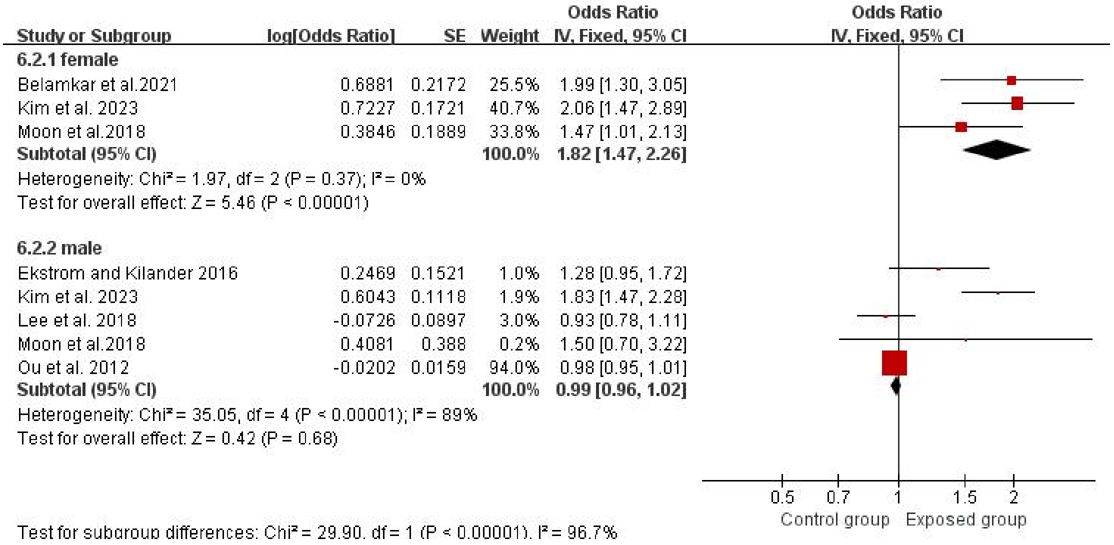

Figure S7. Forest plot showing the effect of gender on Alzheimer's disease with glaucoma

②Age

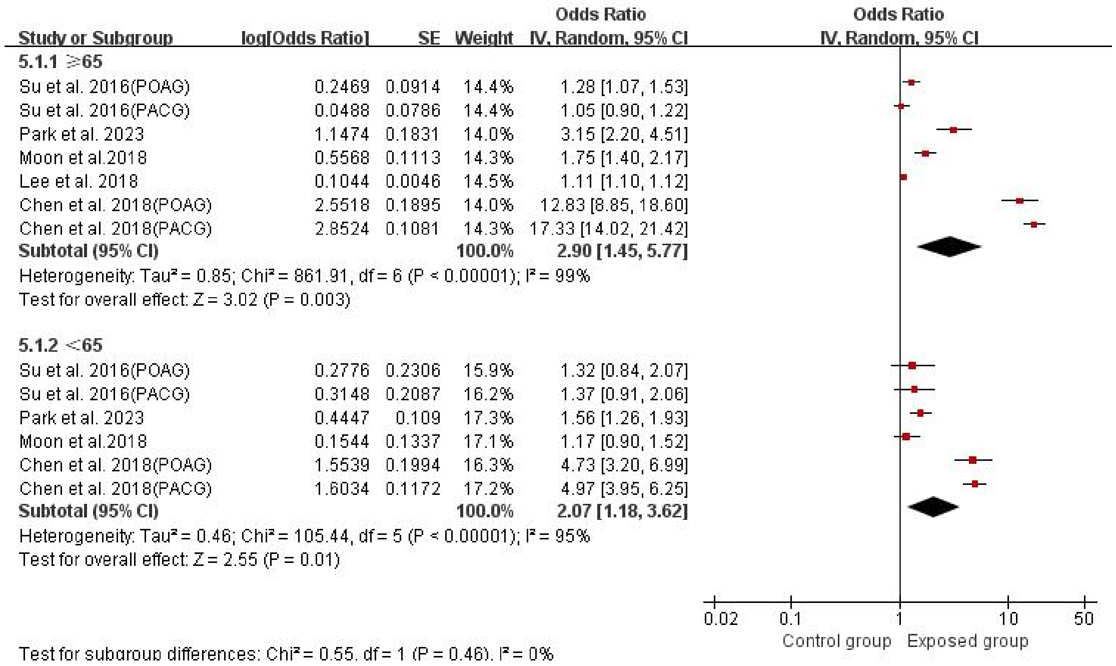

Figure S8. Forest plot showing the effect of age on dementia with glaucoma

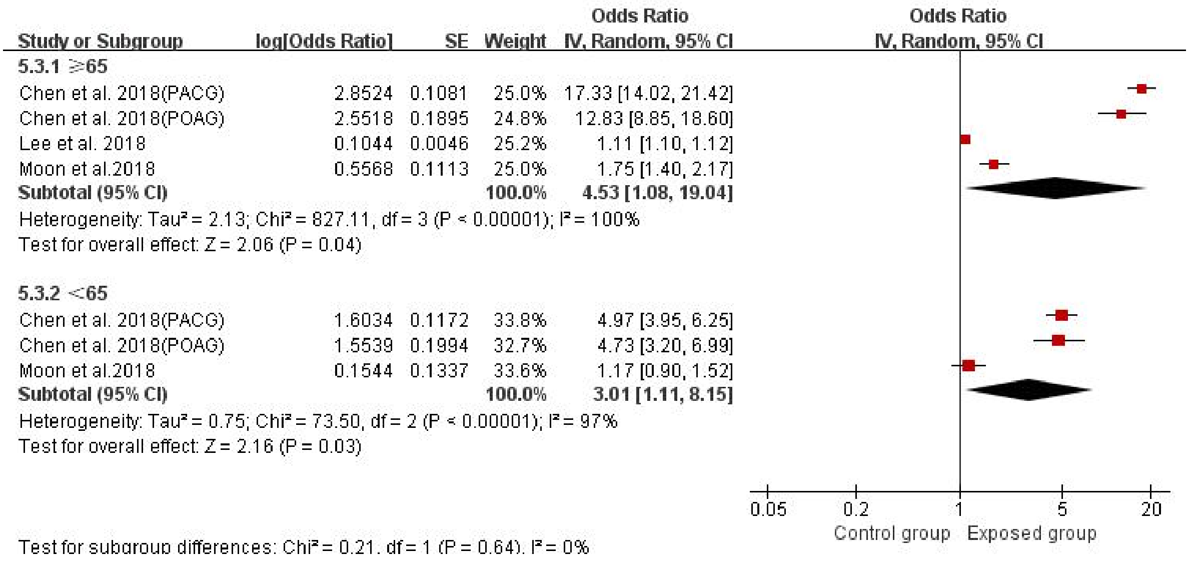

Figure S9. Forest plot showing the effect of age on Alzheimer's disease with glaucoma

Random-effects model

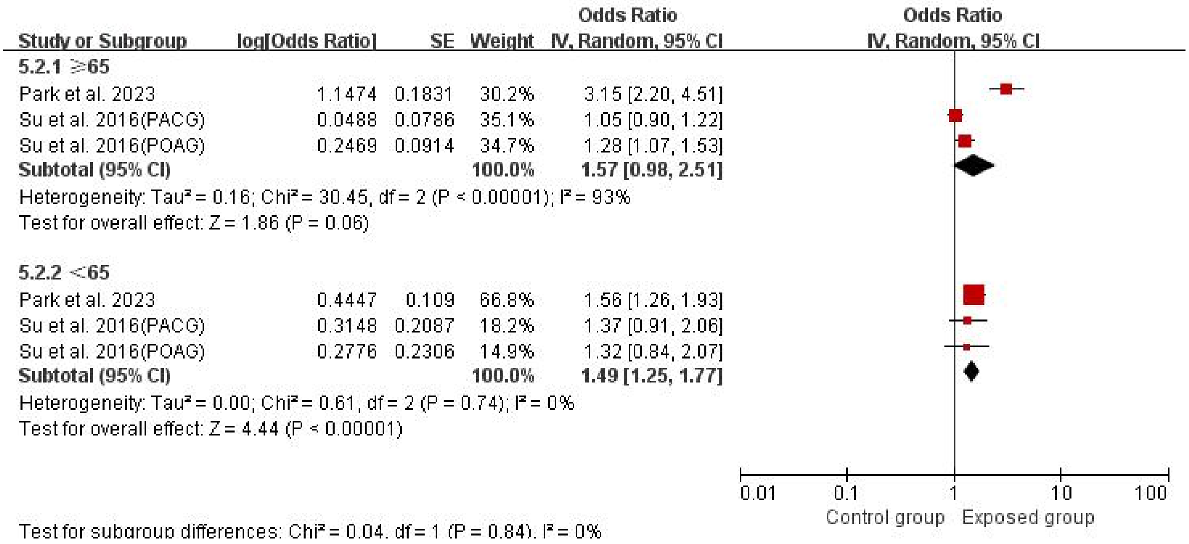

fixed-effects model

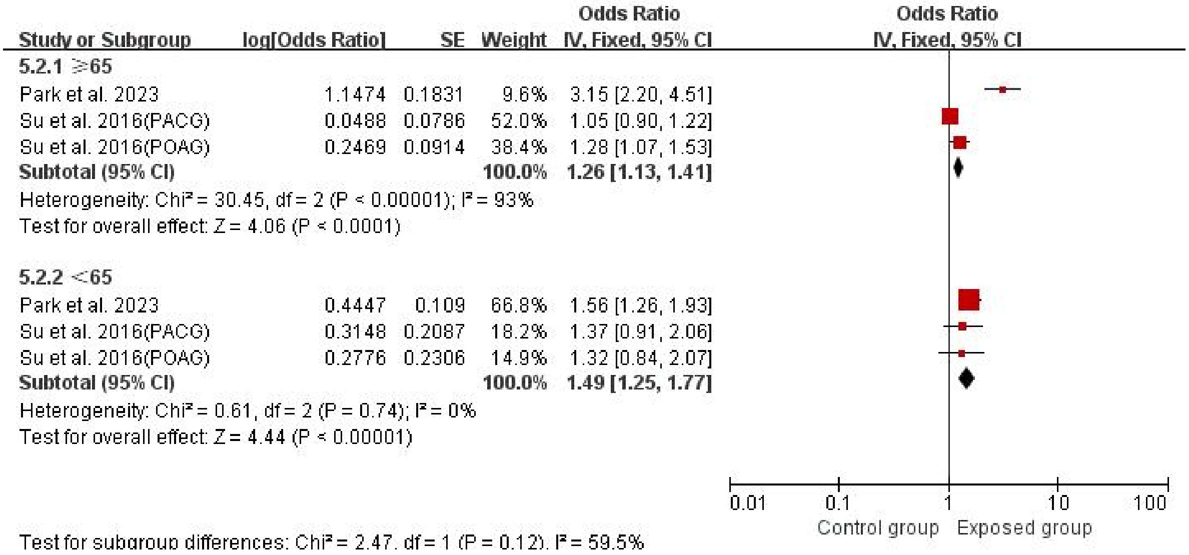

Figure S10. Forest plot showing the effect of age on all-cause dementia with glaucoma

③**Sample size**

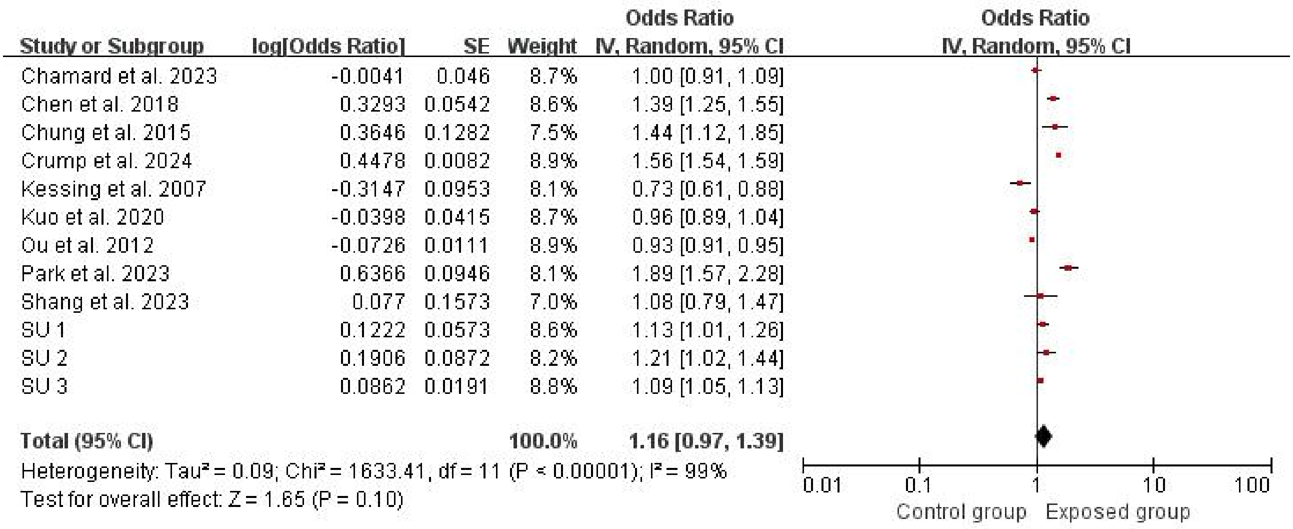

Figure S11. Forest plot showing the effect of sample size≥10000 on all-cause dementia

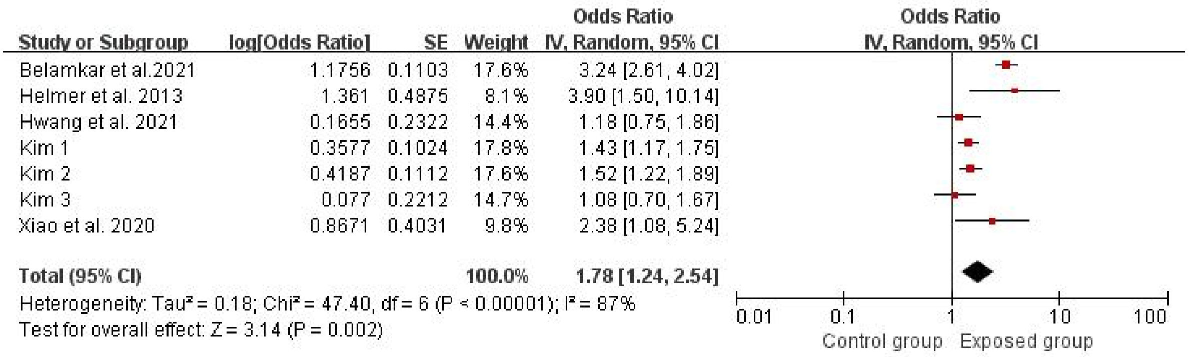

Figure S12. Forest plot showing the effect of sample size＜10000 on all-cause dementia

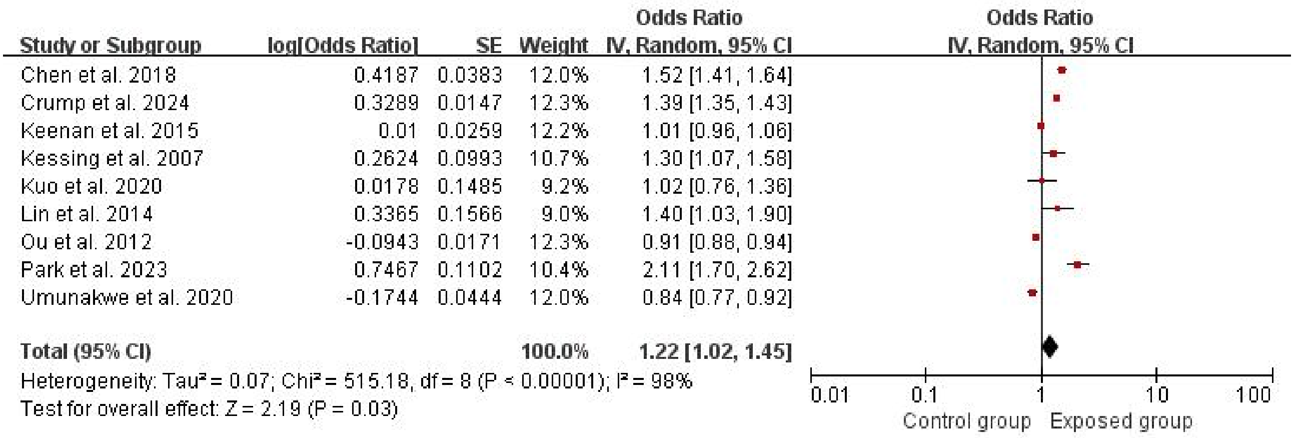

Figure S13. Forest plot showing the effect of sample size≥10000 on Alzheimer's disease

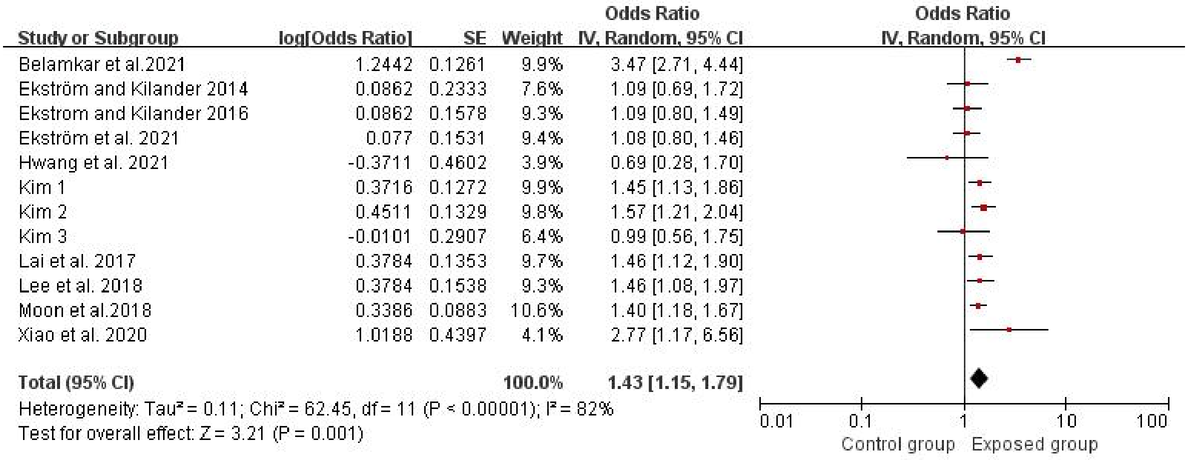

Figure S14. Forest plot showing the effect of sample size＜10000 on Alzheimer's disease

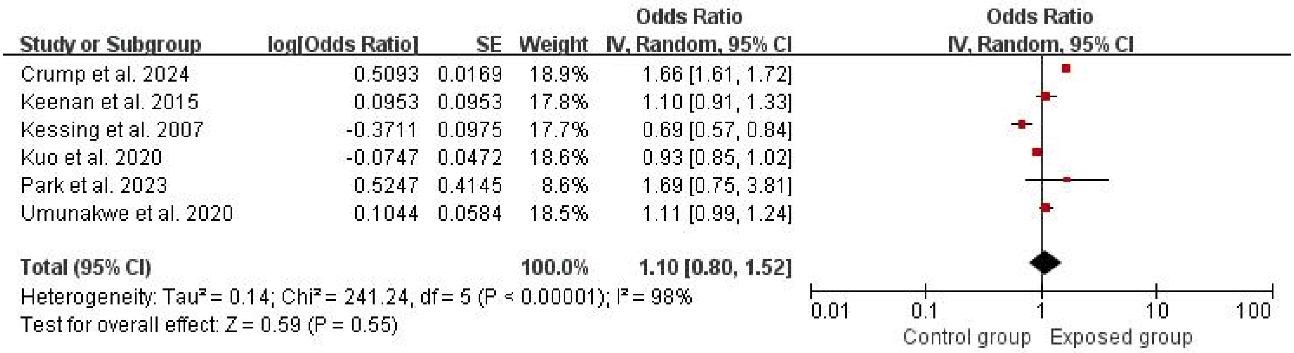

Figure S15. Forest plot showing the effect of sample size≥10000 on vascular dementia

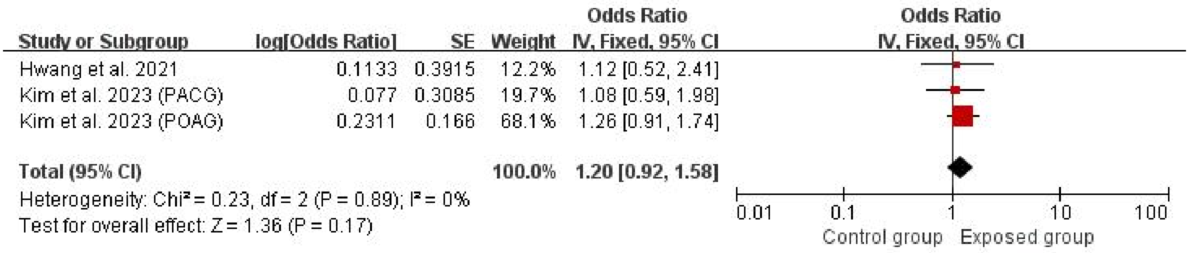

Figure S16. Forest plot showing the effect of sample size＜10000 on vascular dementia

④Mean follow-up time

Study or Subgroup	log[Odds Ratio]	SE	Weight	Odds Ratio IV, Random, 95% CI
Belamkar et al.2021	1.1756	0.1103	11.0%	3.24 [2.61, 4.02]
Kuo et al. 2020	-0.0398	0.0415	13.9%	0.96 [0.89, 1.04]
Ou et al. 2012	-0.0726	0.0111	14.5%	0.93 [0.91, 0.95]
Park et al. 2023	0.6366	0.0946	11.8%	1.89 [1.57, 2.28]
Shang et al. 2023	0.077	0.1573	8.8%	1.08 [0.79, 1.47]
SU 1	0.1222	0.0573	13.4%	1.13 [1.01, 1.26]
SU 2	0.1906	0.0872	12.1%	1.21 [1.02, 1.44]
SU 3	0.0862	0.0191	14.5%	1.09 [1.05, 1.13]
**Total (95% CI)**			**100.0%**	**1.28 [1.11, 1.48]**

Heterogeneity: Tau² = 0.04; Chi² = 230.83, df = 7 (P < 0.00001); I² = 97%
Test for overall effect: Z = 3.35 (P = 0.0008)

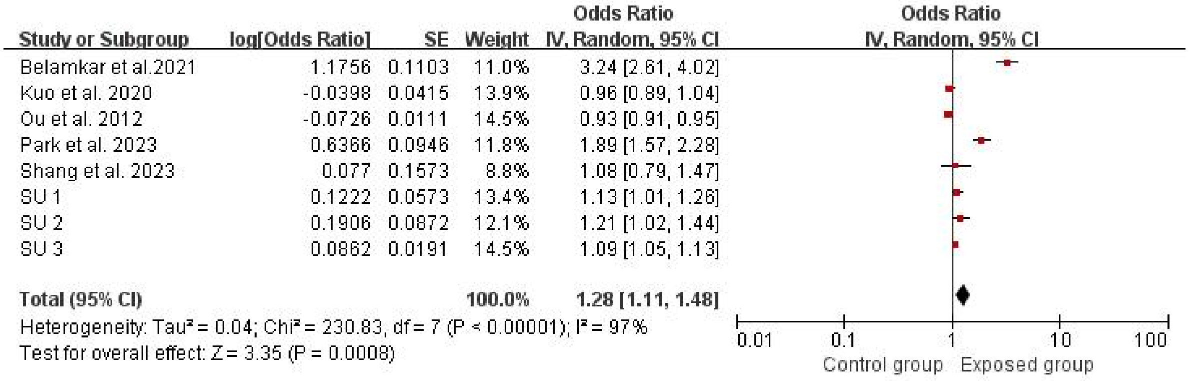

Figure S17. Forest plot showing the effect of follow-up time≥10 years on all-cause dementia

Study or Subgroup	log[Odds Ratio]	SE	Weight	Odds Ratio IV, Random, 95% CI
Chen et al. 2018	0.3293	0.0542	15.5%	1.39 [1.25, 1.55]
Chung et al. 2015	0.3646	0.1282	13.3%	1.44 [1.12, 1.85]
Helmer et al. 2013	1.361	0.4875	4.0%	3.90 [1.50, 10.14]
Hwang et al. 2021	0.1655	0.2322	9.5%	1.18 [0.75, 1.86]
Kessing et al. 2007	-0.3147	0.0953	14.4%	0.73 [0.61, 0.88]
Kim 1	0.3577	0.1024	14.2%	1.43 [1.17, 1.75]
Kim 2	0.4187	0.1112	13.9%	1.52 [1.22, 1.89]
Kim 3	0.077	0.2212	9.9%	1.08 [0.70, 1.67]
Xiao et al. 2020	0.8671	0.4031	5.2%	2.38 [1.08, 5.24]
**Total (95% CI)**			**100.0%**	**1.33 [1.07, 1.66]**

Heterogeneity: Tau² = 0.08; Chi² = 49.74, df = 8 (P < 0.00001); I² = 84%
Test for overall effect: Z = 2.55 (P = 0.01)

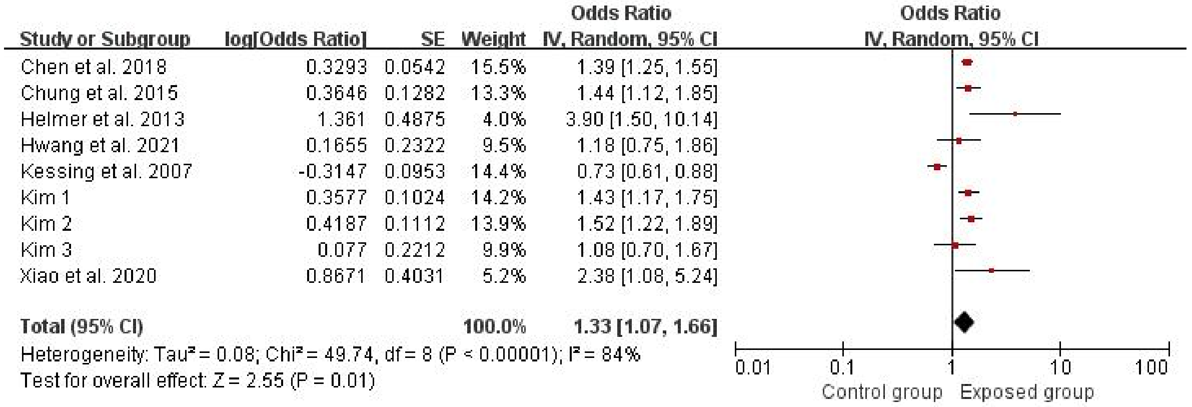

Figure S18. Forest plot showing the effect of follow-up time<10 years on all-cause dementia

Study or Subgroup	log[Odds Ratio]	SE	Weight	Odds Ratio IV, Random, 95% CI
Belamkar et al.2021	1.2442	0.1261	9.1%	3.47 [2.71, 4.44]
Ekström and Kilander 2014	0.0862	0.2333	6.2%	1.09 [0.69, 1.72]
Ekstrom and Kilander 2016	0.0862	0.1578	8.2%	1.09 [0.80, 1.49]
Ekström et al. 2021	0.077	0.1531	8.3%	1.08 [0.80, 1.46]
Keenan et al. 2015	0.01	0.0259	11.1%	1.01 [0.96, 1.06]
Kuo et al. 2020	0.0178	0.1485	8.5%	1.02 [0.76, 1.36]
Lai 1	0.3853	0.1342	8.9%	1.47 [1.13, 1.91]
Lai 2	0.3784	0.1353	8.8%	1.46 [1.12, 1.90]
Moon et al.2018	0.3386	0.0883	10.1%	1.40 [1.18, 1.67]
Ou et al. 2012	-0.0943	0.0171	11.2%	0.91 [0.88, 0.94]
Park et al. 2023	0.7467	0.1102	9.5%	2.11 [1.70, 2.62]
**Total (95% CI)**			**100.0%**	**1.35 [1.13, 1.60]**

Heterogeneity: Tau² = 0.07; Chi² = 204.31, df = 10 (P < 0.00001); I² = 95%
Test for overall effect: Z = 3.41 (P = 0.0007)

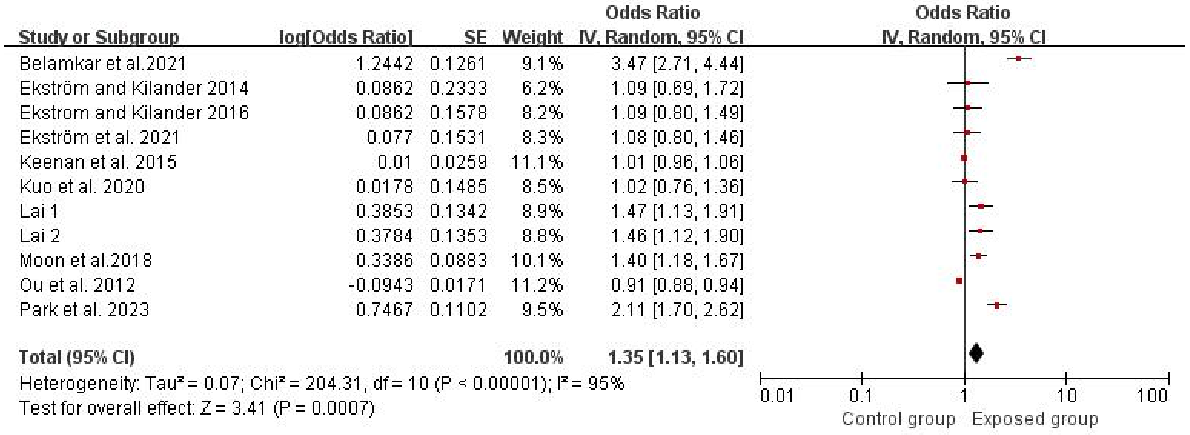

Figure S19. Forest plot showing the effect of follow-up time≥10 years on Alzheimer's disease

Study or Subgroup	log[Odds Ratio]	SE	Weight	Odds Ratio IV, Random, 95% CI
Chen et al. 2018	0.4187	0.0383	13.2%	1.52 [1.41, 1.64]
Hwang et al. 2021	-0.3711	0.4602	4.5%	0.69 [0.28, 1.70]
Kessing et al. 2007	0.2624	0.0993	12.2%	1.30 [1.07, 1.58]
Kim 1	0.3716	0.1272	11.6%	1.45 [1.13, 1.86]
Kim 2	0.4511	0.1329	11.5%	1.57 [1.21, 2.04]
Kim 3	-0.0101	0.2907	7.4%	0.99 [0.56, 1.75]
Lee et al. 2018	0.3784	0.1538	10.9%	1.46 [1.08, 1.97]
Lin et al. 2014	0.3365	0.1566	10.9%	1.40 [1.03, 1.90]
Umunakwe et al. 2020	-0.1744	0.0444	13.1%	0.84 [0.77, 0.92]
Xiao et al. 2020	1.0188	0.4397	4.7%	2.77 [1.17, 6.56]
**Total (95% CI)**			**100.0%**	**1.31 [1.04, 1.65]**

Heterogeneity: Tau² = 0.11; Chi² = 117.13, df = 9 (P < 0.00001); I² = 92%
Test for overall effect: Z = 2.25 (P = 0.02)

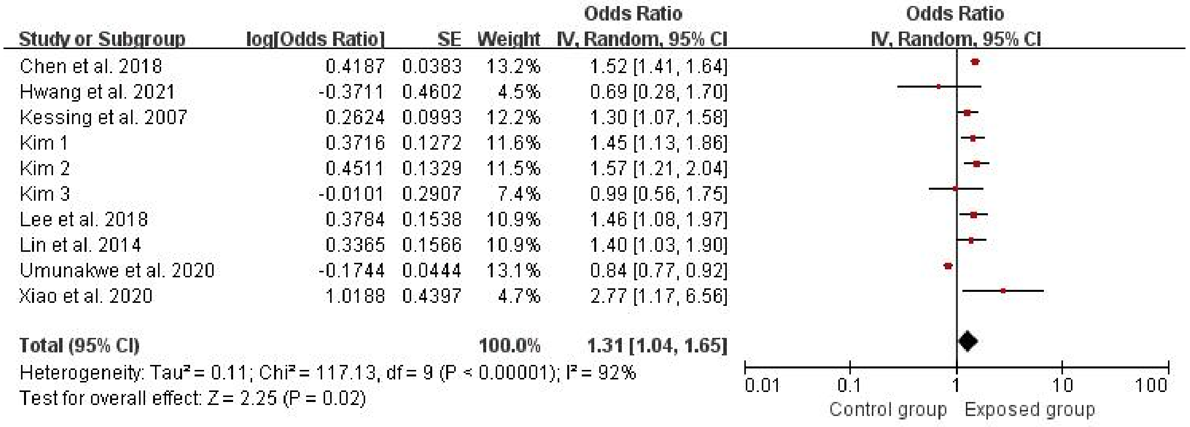

Figure S20. Forest plot showing the effect of follow-up time＜10 years on Alzheimer's disease

Study or Subgroup	log[Odds Ratio]	SE	Weight	Odds Ratio IV, Random, 95% CI
Keenan et al. 2015	0.0953	0.0953	38.9%	1.10 [0.91, 1.33]
Kuo et al. 2020	-0.0747	0.0472	56.5%	0.93 [0.85, 1.02]
Park et al. 2023	0.5247	0.4145	4.6%	1.69 [0.75, 3.81]
**Total (95% CI)**			**100.0%**	**1.02 [0.85, 1.22]**

Heterogeneity: Tau² = 0.01; Chi² = 4.40, df = 2 (P = 0.11); I² = 55%
Test for overall effect: Z = 0.21 (P = 0.84)

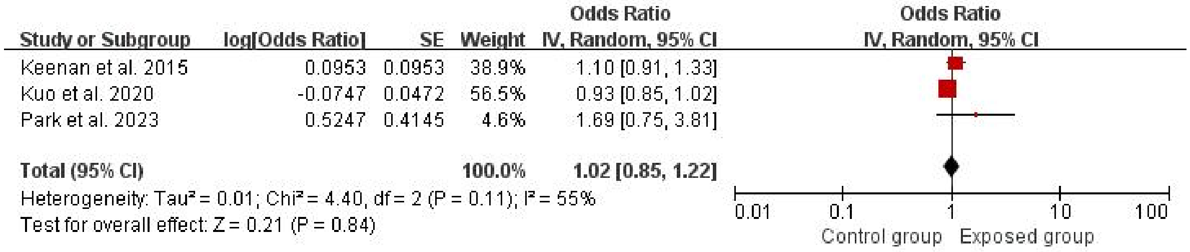

Figure S21. Forest plot showing the effect of follow-up time≥10 years on vascular dementia

Study or Subgroup	log[Odds Ratio]	SE	Weight	Odds Ratio IV, Random, 95% CI
Hwang et al. 2021	0.1133	0.3915	9.4%	1.12 [0.52, 2.41]
Kessing et al. 2007	-0.3711	0.0975	26.9%	0.69 [0.57, 0.84]
Kim et al. 2023 (PACG)	0.077	0.3085	12.7%	1.08 [0.59, 1.98]
Kim et al. 2023 (POAG)	0.2311	0.166	21.8%	1.26 [0.91, 1.74]
Umunakwe et al. 2020	0.1044	0.0584	29.2%	1.11 [0.99, 1.24]
**Total (95% CI)**			**100.0%**	**1.00 [0.76, 1.33]**

Heterogeneity: Tau² = 0.07; Chi² = 19.76, df = 4 (P = 0.0006); I² = 80%
Test for overall effect: Z = 0.01 (P = 0.99)

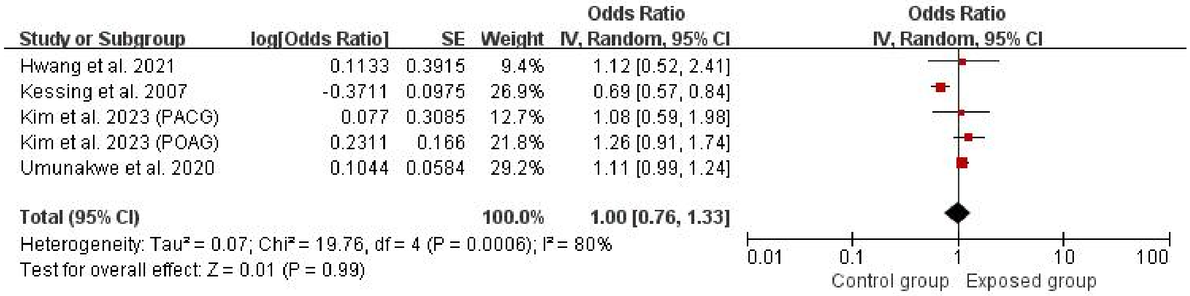

Figure S22. Forest plot showing the effect of follow-up time＜10 years on vascular dementia

⑤Geographic location

Study or Subgroup	log[Odds Ratio]	SE	Weight	Odds Ratio IV, Random, 95% CI
Chen et al. 2018	0.3293	0.0542	11.8%	1.39 [1.25, 1.55]
Chung et al. 2015	0.3646	0.1282	8.1%	1.44 [1.12, 1.85]
Kim 1	0.3577	0.1024	9.4%	1.43 [1.17, 1.75]
Kim 2	0.4187	0.1112	9.0%	1.52 [1.22, 1.89]
Kim 3	0.077	0.2212	4.6%	1.08 [0.70, 1.67]
Kuo et al. 2020	-0.0398	0.0415	12.3%	0.96 [0.89, 1.04]
Park et al. 2023	0.6366	0.0946	9.8%	1.89 [1.57, 2.28]
SU 1	0.1222	0.0573	11.7%	1.13 [1.01, 1.26]
SU 2	0.1906	0.0872	10.2%	1.21 [1.02, 1.44]
SU 3	0.0862	0.0191	13.0%	1.09 [1.05, 1.13]
Xiao et al. 2020	0.8671	0.4031	0.0%	2.38 [1.08, 5.24]
**Total (95% CI)**			**100.0%**	**1.28 [1.14, 1.44]**

Heterogeneity: Tau² = 0.03; Chi² = 79.59, df = 9 (P < 0.00001); I² = 89%
Test for overall effect: Z = 4.16 (P < 0.0001)

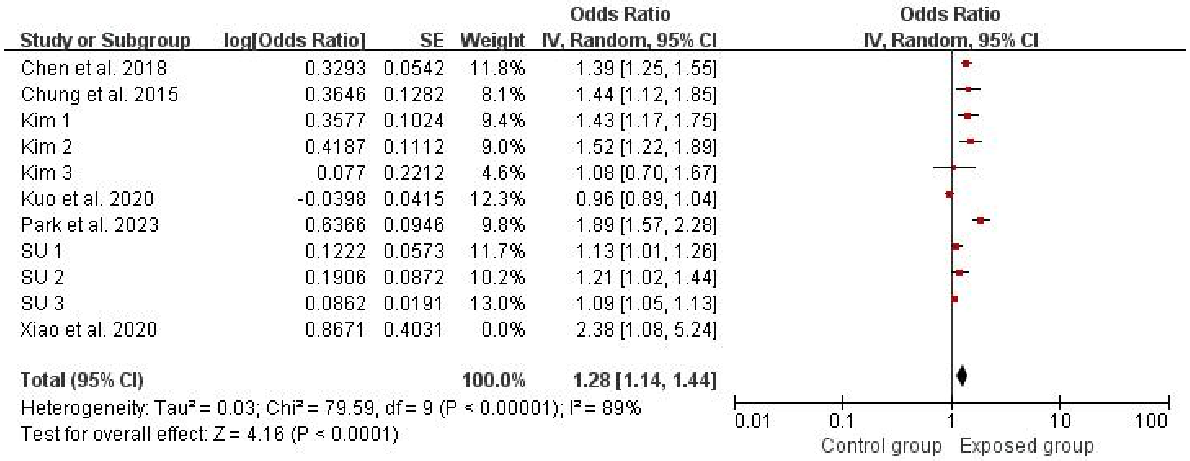

Figure S23. Forest plot showing the effect of glaucoma on all-cause dementia in Asia

Study or Subgroup	log[Odds Ratio]	SE	Weight	Odds Ratio IV, Random, 95% CI
Chamard et al. 2023	-0.0041	0.046	23.9%	1.00 [0.91, 1.09]
Crump et al. 2024	0.4478	0.0082	24.3%	1.56 [1.54, 1.59]
Helmer et al. 2013	1.361	0.4875	8.6%	3.90 [1.50, 10.14]
Kessing et al. 2007	-0.3147	0.0953	22.7%	0.73 [0.61, 0.88]
Shang et al. 2023	0.077	0.1573	20.4%	1.08 [0.79, 1.47]
**Total (95% CI)**			**100.0%**	**1.18 [0.83, 1.68]**

Heterogeneity: Tau² = 0.13; Chi² = 163.44, df = 4 (P < 0.00001); I² = 98%
Test for overall effect: Z = 0.95 (P = 0.34)

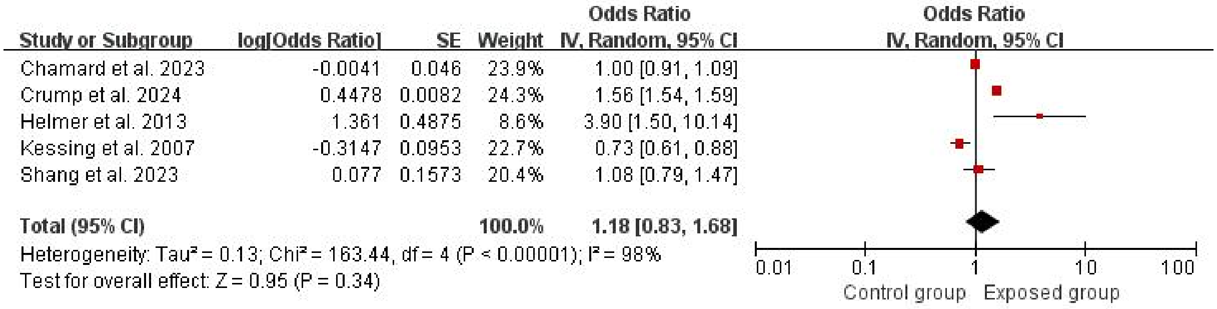

Figure S24. Forest plot showing the effect of glaucoma on all-cause dementia in Europe

Study or Subgroup	log[Odds Ratio]	SE	Weight	Odds Ratio IV, Fixed, 95% CI
Belamkar et al.2021	1.1756	0.1103	1.0%	3.24 [2.61, 4.02]
Hwang et al. 2021	0.1655	0.2322	0.2%	1.18 [0.75, 1.86]
Ou et al. 2012	-0.0726	0.0111	98.8%	0.93 [0.91, 0.95]
**Total (95% CI)**			**100.0%**	**0.94 [0.92, 0.96]**

Heterogeneity: Chi² = 127.72, df = 2 (P < 0.00001); I² = 98%
Test for overall effect: Z = 5.40 (P < 0.00001)

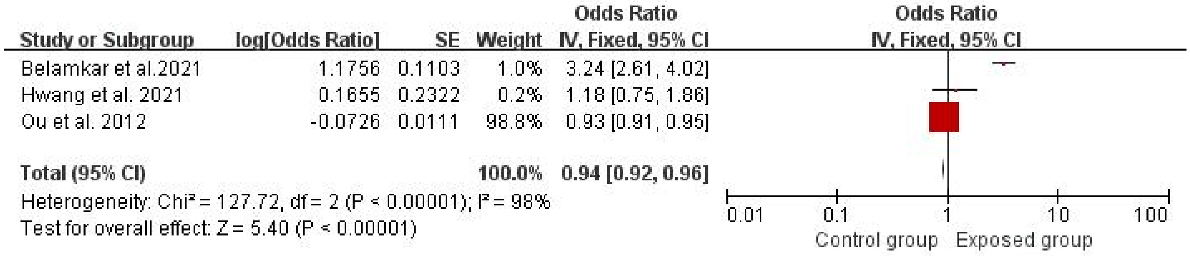

Figure S25. Forest plot showing the effect of glaucoma on all-cause dementia in North America

Study or Subgroup	log[Odds Ratio]	SE	Weight	Odds Ratio IV, Random, 95% CI
Chen et al. 2018	0.4187	0.0383	18.7%	1.52 [1.41, 1.64]
Kim 1	0.3716	0.1272	9.6%	1.45 [1.13, 1.86]
Kim 2	0.4511	0.1329	9.2%	1.57 [1.21, 2.04]
Kim 3	-0.0101	0.2907	3.0%	0.99 [0.56, 1.75]
Kuo et al. 2020	0.0178	0.1485	8.1%	1.02 [0.76, 1.36]
Lai 1	0.3853	0.1342	9.1%	1.47 [1.13, 1.91]
Lai 2	0.3784	0.1353	9.0%	1.46 [1.12, 1.90]
Lin et al. 2014	0.3365	0.1566	7.6%	1.40 [1.03, 1.90]
Moon et al.2018	0.3386	0.0883	13.3%	1.40 [1.18, 1.67]
Park et al. 2023	0.7467	0.1102	11.1%	2.11 [1.70, 2.62]
Xiao et al. 2020	1.0188	0.4397	1.4%	2.77 [1.17, 6.56]
**Total (95% CI)**			**100.0%**	**1.48 [1.33, 1.65]**

Heterogeneity: Tau² = 0.01; Chi² = 21.52, df = 10 (P = 0.02); I² = 54%
Test for overall effect: Z = 7.27 (P < 0.00001)

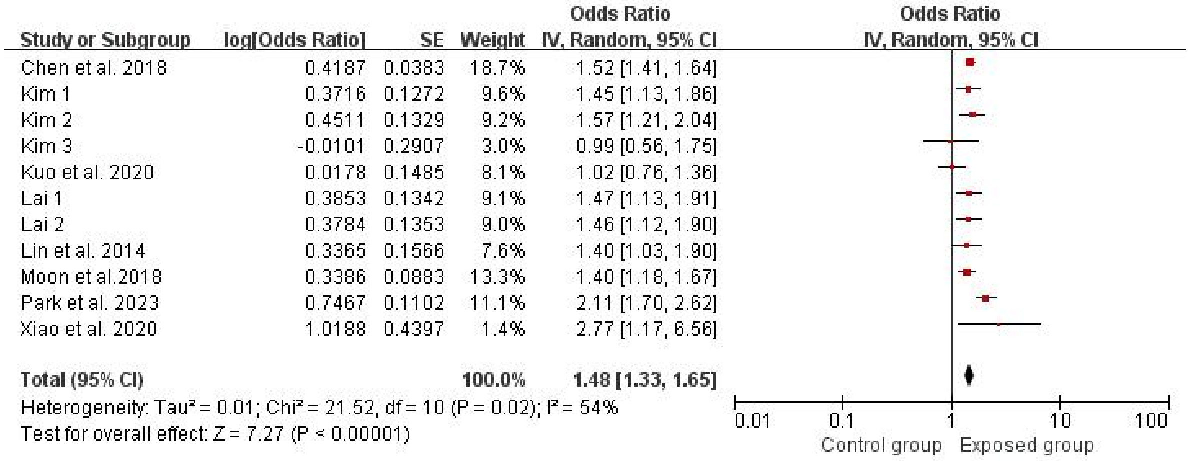

Figure S26. Forest plot showing the effect of glaucoma on Alzheimer's disease in Asia

Study or Subgroup	log[Odds Ratio]	SE	Weight	Odds Ratio IV, Random, 95% CI
Crump et al. 2024	0.3289	0.0147	22.0%	1.39 [1.35, 1.43]
Ekström and Kilander 2014	0.0862	0.2333	9.8%	1.09 [0.69, 1.72]
Ekstrom and Kilander 2016	0.0862	0.1578	14.1%	1.09 [0.80, 1.49]
Ekström et al. 2021	0.077	0.1531	14.4%	1.08 [0.80, 1.46]
Keenan et al. 2015	0.01	0.0259	21.8%	1.01 [0.96, 1.06]
Kessing et al. 2007	0.2624	0.0993	18.0%	1.30 [1.07, 1.58]
**Total (95% CI)**			**100.0%**	**1.17 [0.96, 1.41]**

Heterogeneity: Tau² = 0.04; Chi² = 117.53, df = 5 (P < 0.00001); I² = 96%
Test for overall effect: Z = 1.56 (P = 0.12)

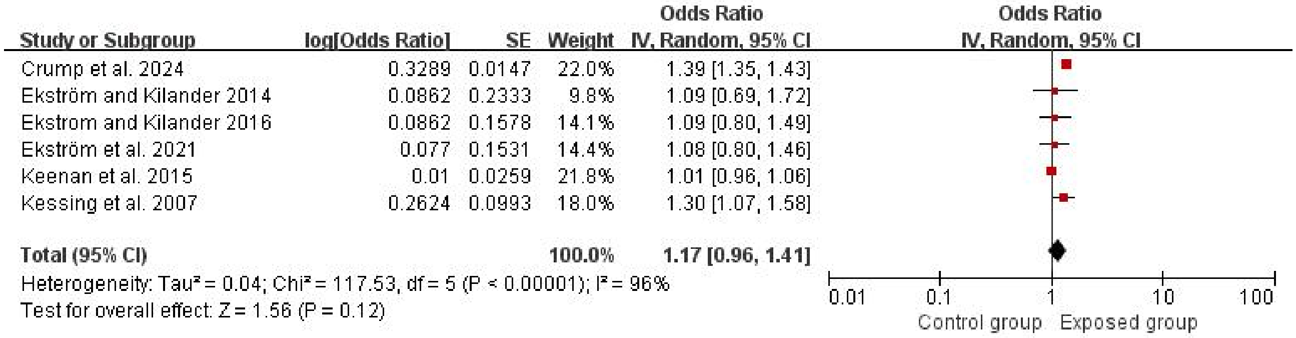

Figure S27. Forest plot showing the effect of glaucoma on Alzheimer's disease in Europe

Study or Subgroup	log[Odds Ratio]	SE	Weight	Odds Ratio IV, Random, 95% CI
Belamkar et al.2021	1.2442	0.1261	21.7%	3.47 [2.71, 4.44]
Hwang et al. 2021	-0.3711	0.4602	8.6%	0.69 [0.28, 1.70]
Lee et al. 2018	0.3784	0.1538	20.5%	1.46 [1.08, 1.97]
Ou et al. 2012	-0.0943	0.0171	24.8%	0.91 [0.88, 0.94]
Umunakwe et al. 2020	-0.1744	0.0444	24.4%	0.84 [0.77, 0.92]
**Total (95% CI)**			**100.0%**	**1.28 [0.93, 1.78]**

Heterogeneity: Tau² = 0.11; Chi² = 124.77, df = 4 (P < 0.00001); I² = 97%
Test for overall effect: Z = 1.50 (P = 0.13)

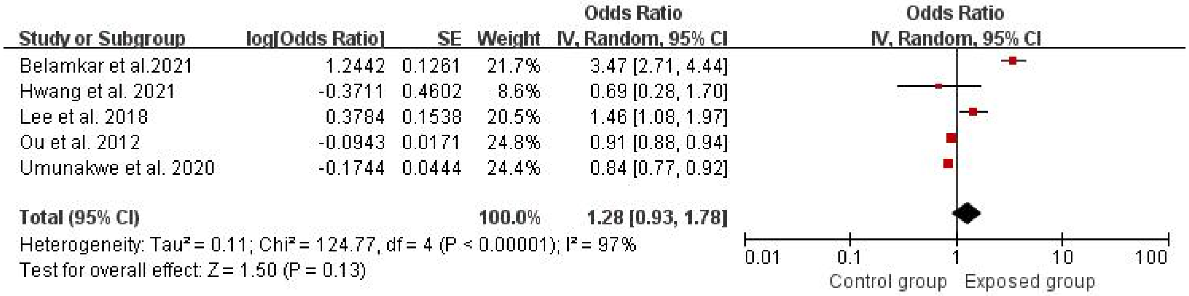

Figure S28. Forest plot showing the effect of glaucoma on Alzheimer's disease in North America

Study or Subgroup	log[Odds Ratio]	SE	Weight	Odds Ratio IV, Fixed, 95% CI
Kim 2	0.2311	0.166	7.2%	1.26 [0.91, 1.74]
Kim 3	0.077	0.3085	2.1%	1.08 [0.59, 1.98]
Kuo et al. 2020	-0.0747	0.0472	89.5%	0.93 [0.85, 1.02]
Park et al. 2023	0.5247	0.4145	1.2%	1.69 [0.75, 3.81]
**Total (95% CI)**			**100.0%**	**0.96 [0.88, 1.05]**

Heterogeneity: Chi² = 5.20, df = 3 (P = 0.16); I² = 42%
Test for overall effect: Z = 0.95 (P = 0.34)

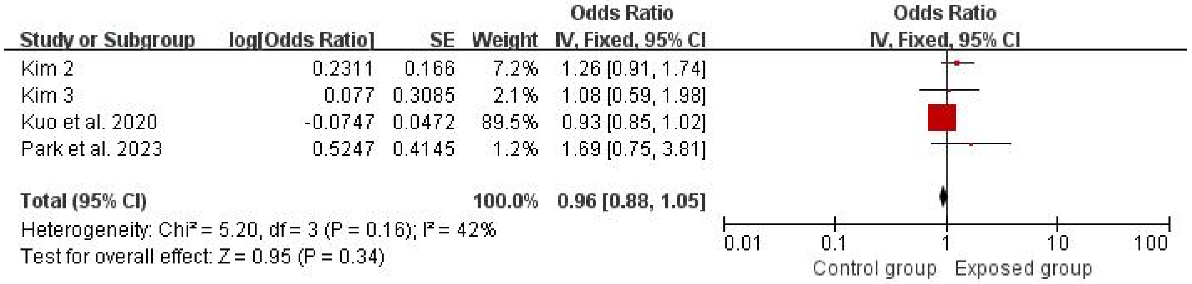

Figure S29. Forest plot showing the effect of glaucoma on vascular dementia in Asia

Study or Subgroup	log[Odds Ratio]	SE	Weight	Odds Ratio IV, Random, 95% CI
Crump et al. 2024	0.5093	0.0169	34.2%	1.66 [1.61, 1.72]
Keenan et al. 2015	0.0953	0.0953	32.9%	1.10 [0.91, 1.33]
Kessing et al. 2007	-0.3711	0.0975	32.9%	0.69 [0.57, 0.84]
**Total (95% CI)**			**100.0%**	**1.09 [0.63, 1.88]**

Heterogeneity: Tau² = 0.23; Chi² = 95.27, df = 2 (P < 0.00001); I² = 98%
Test for overall effect: Z = 0.30 (P = 0.76)

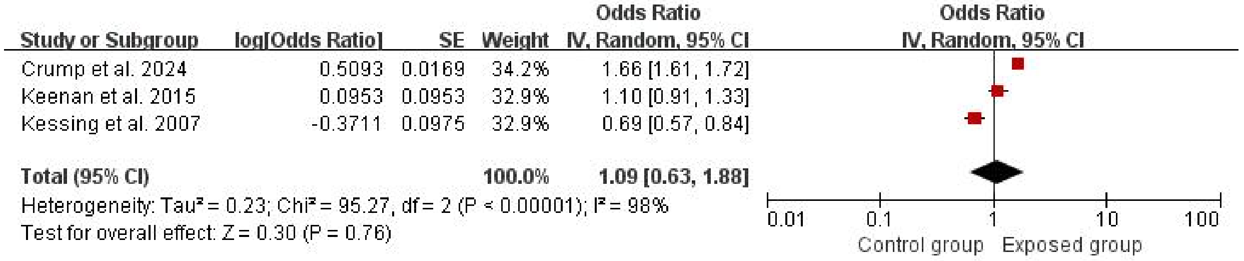

Figure S30. Forest plot showing the effect of glaucoma on vascular dementia in Europe

Study or Subgroup	log[Odds Ratio]	SE	Weight	Odds Ratio IV, Fixed, 95% CI
Hwang et al. 2021	0.1133	0.3915	2.2%	1.12 [0.52, 2.41]
Umunakwe et al. 2020	0.1044	0.0584	97.8%	1.11 [0.99, 1.24]
**Total (95% CI)**			**100.0%**	**1.11 [0.99, 1.24]**

Heterogeneity: Chi² = 0.00, df = 1 (P = 0.98); I² = 0%
Test for overall effect: Z = 1.81 (P = 0.07)

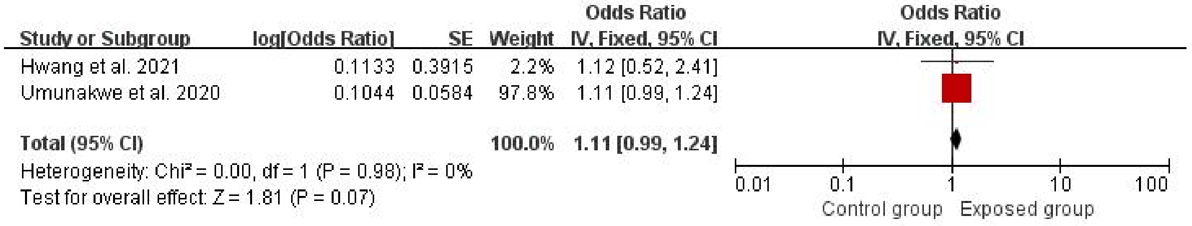

Figure S31. Forest plot showing the effect of glaucoma on vascular dementia in North America
