# Supplementary material for: Risk of glaucoma to subsequent dementia or cognitive impairment: a systematic review and meta-analysis
Source: Aging Clin Exp Res. 2024 Aug 20;36(1):172. doi: 10.1007/s40520-024-02811-w (PMC11335947; doi:10.1007/s40520-024-02811-w)
Supplement: Supplementary file 6 — Supplementary Material 6 [file 40520_2024_2811_MOESM6_ESM.pdf]

Supplementary Appendix

Supplementary Appendix 1: *PRISMA Checklist*

Supplementary Appendix 2: Details of the Literature Search Strategy

Supplementary Appendix 3: Excluded studies after reading the full text

Supplementary Appendix 4: Sensitivity Analysis

Supplementary Appendix 5: Subgroup analysis
